# Angiotensin type 1 receptor activation promotes neuronal and glial alpha-synuclein aggregation and transmission

**DOI:** 10.1038/s41531-024-00650-0

**Published:** 2024-02-17

**Authors:** Lucia Lage, Ana I. Rodriguez-Perez, Begoña Villar-Cheda, Jose L. Labandeira-Garcia, Antonio Dominguez-Meijide

**Affiliations:** 1 Cellular and Molecular Neurobiology of Parkinson’s disease, Research Center for Molecular Medicine and Chronic diseases (CIMUS), IDIS, University of Santiago de Compostela, Santiago de Compostela, Spain; 2 Networking Research Center on Neurodegenerative Diseases (CIBERNED), Madrid, Spain

**Keywords:** Parkinson's disease, Experimental models of disease

## Abstract

The brain renin-angiotensin system (RAS) has been related to dopaminergic degeneration, and high expression of the angiotensin II (AngII) type 1 receptor (AT1) gene is a marker of the most vulnerable neurons in humans. However, it is unknown whether AngII/AT1 overactivation affects α-synuclein aggregation and transmission. In vitro, AngII/AT1 activation increased α-synuclein aggregation in dopaminergic neurons and microglial cells, which was related to AngII-induced NADPH-oxidase activation and intracellular calcium raising. In mice, AngII/AT1 activation was involved in MPTP-induced increase in α-synuclein expression and aggregation, as they significantly decreased in mice treated with the AT1 blocker telmisartan and AT1 knockout mice. Cell co-cultures (transwells) revealed strong transmission of α-synuclein from dopaminergic neurons to astrocytes and microglia. AngII induced a higher α-synuclein uptake by microglial cells and an increase in the transfer of α-synuclein among astroglial cells. However, AngII did not increase the release of α-synuclein by neurons. The results further support brain RAS dysregulation as a major mechanism for the progression of Parkinson’s disease, and AT1 inhibition and RAS modulation as therapeutic targets.

## Introduction

Parkinson’s disease (PD) is the second most common neurodegenerative disease after Alzheimer’s disease, and its prevalence is increasing related to the increase in the aged population^1^. The pathophysiology of PD has not been totally clarified. Several major processes are known to be involved in dopaminergic degeneration, although the reason for initiating these processes and their order of appearance are still unclear. Major mechanisms involved in the progression of the disease are oxidative stress, neuroinflammation, and the presence of protein aggregates termed Lewy bodies (LBs). One of the characteristics of these aggregates is the presence of misfolded forms of α-synuclein^2^. The α-synuclein aggregates may spread through the brain, propagating the disease^3^. This progression may happen through a cell-to-cell transmission by different mechanisms. It has been proposed that cell-to-cell transmission of α-synuclein happens preferentially between neurons and from neurons to astrocytes^4^, and that astrocytes and microglial cells may have some effect in regulating cell-to-cell transfer of α-synuclein^5–7^.

Previous studies from our group and others have related the brain renin-angiotensin system (RAS) to the progression of dopaminergic neurodegeneration in several animal models^8–10^. The presence of a local RAS in the substantia nigra and striatum of rodents, non-human and human primates has also been shown^11,12^. This was further supported by a recent study using single‐cell genomic profiling of human dopaminergic neurons that identified a high expression of angiotensin type 1 receptor (AT1) gene as a marker of the most vulnerable dopaminergic neurons in humans, including PD patients^13^, and by recent clinical studies showing neuroprotective effects of AT1 receptor blockers on PD risk^14,15^. Astrocytes are the major source of the angiotensin precursor protein angiotensinogen and paracrine or tissular angiotensin II (AngII, the main effector peptide of the RAS) in the CNS (central nervous system)^16,17^. However, the production of low levels of intraneuronal AngII and an intraneuronal intracrine RAS have also been observed^18^.

Overactivation of the AngII/AT1 axis in neurons and glial cells promotes major processes involved in PD progression, such as oxidative stress by activation of the NADPH-oxidase complex (Nox; the second most relevant source of cellular ROS after the mitochondria), leading to further increase in ROS production from mitochondria via Mitochondrial ATP-sensitive potassium channels^19,20^. Furthermore, AngII/AT1 activation promotes intraneuronal calcium raising^21–23^, and microglial neuroinflammatory responses^24–26^. However, it is unknown whether AngII/AT1 overactivation affects α-synuclein aggregation and transmission in neurons and glial cells.

In the present study, we used in vitro and in vivo models to study the possible effects of AngII on α-synuclein aggregation and transmission in dopaminergic neurons, and astroglial and microglial cells. In vitro, we used the α-synuclein-T/V5-synphilin-1 (SynT/Sph1) and a modified version of the bimolecular fluorescence complementation (BiFC) model. In vivo, we used a subacute MPTP PD mouse model. There was some controversy on α-synuclein aggregation in the MPTP mouse model, as several studies were unable to observe the presence of α-synuclein aggregates in MPTP-treated mice^27,28^, and other studies have reported aggregates^29,30^. These controversial results may be related to the course and doses used in different experiments^31^. We also used the present experiments to clarify this issue.

## Results

### Validation of in vitro models: inclusions with aggregate-like structures were present in neuronal, microglial, and astroglial cells

**Fig. 1:**
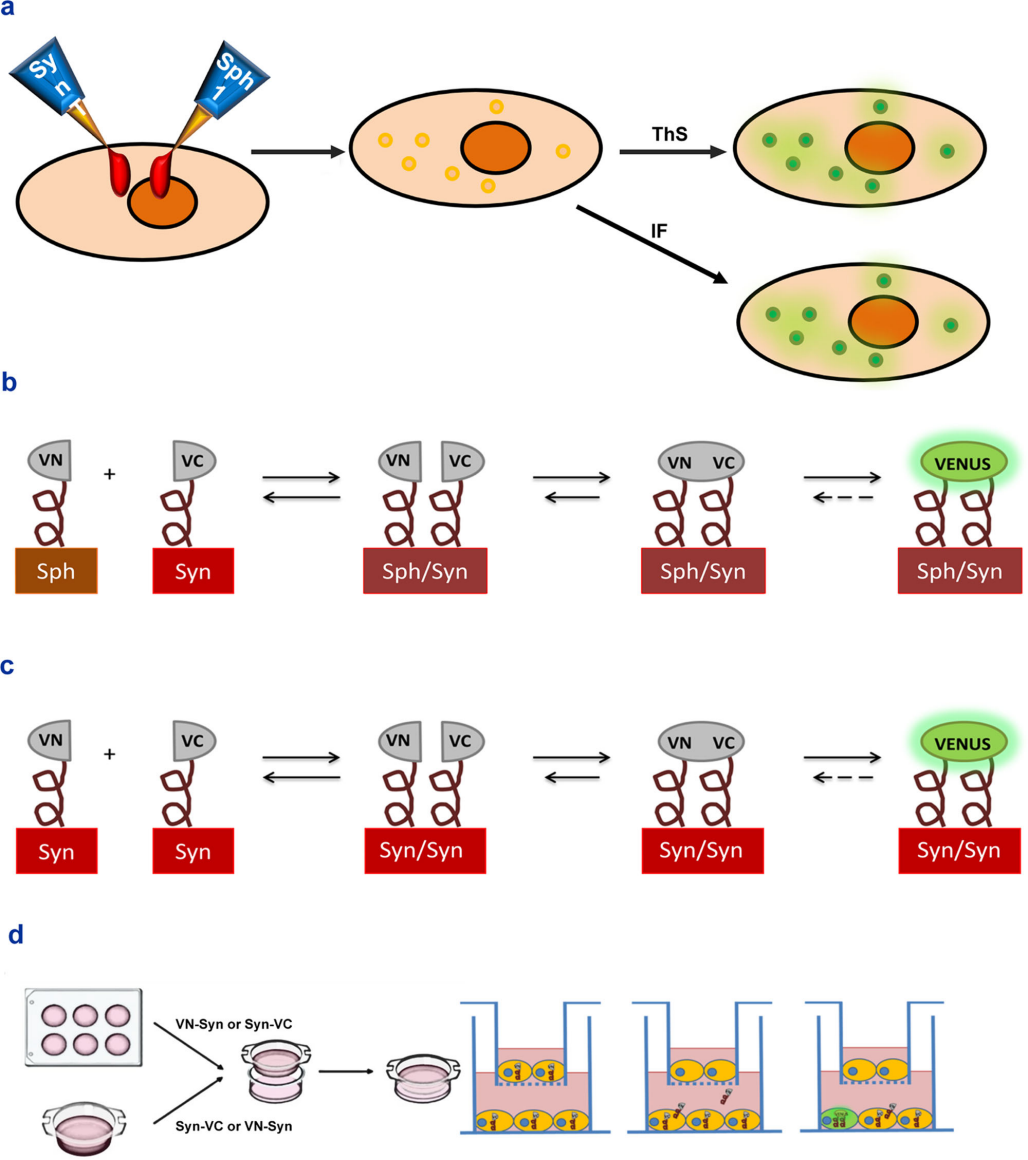
Experimental design and schematic representation of the in vitro experiments. **a** α-synuclein-T/Synphilin-1 method and thioflavin S staining. Concomitant expression of SynT and V5-Sph-1 leads to the formation of inclusions that can be observed by immunofluorescence. The binding of thioflavin S to aggregated α-synuclein leads to fluorescence emission, which was used for qualitative characterization of the inclusions (Fig. 2a), detergent solubilization, SDD-Tricine and native WB (Fig. 2b–h), and quantitative assessment of α-synuclein aggregation (Fig. 3a, b, e, f, i, j, m, o, q, s, u; 5e, g; s1). **b** Synphilin-1-VN/α-synuclein-VC BiFC method. Synphilin-1 tagged to the VN fragment of venus interacts with α-synuclein tagged to the VC fragment of venus, leading to the reconstitution of the fluorophore and the emission of fluorescence, used for quantitative assessment of α-synuclein inclusion size (Fig. 3c, d, g, h, k, l, n, p, r, t, v; 5f, h; s1). **c** VN-α-synuclein/α-synuclein-VC BiFC technique. α-synuclein tagged to the VN fragment of venus interacts with α-synuclein tagged to the VC fragment of venus leading to fluorescence, used for detergent solubilization, SDS-PAGE, SDD-Tricine and native WB (Fig. 2b–h; 4a–h), and assessment of transfection levels by flow cytometry (Fig. 4i–l; s3–s4). **d** Cell-to-cell transfer of α-synuclein. Cells are cultured in culture plates and transwells and transfected, each with different halves of the BiFC system. Transwells are inserted into culture plate wells. Tagged α-synuclein migrates from the cells inside the transwells to the cells inside the plate and vice versa, forming dimers, oligomers, or aggregates that may reconstitute the fluorescence of the BiFC system that is measured by flow cytometry (Fig. 7, 8, s5–s7). IF: immunofluorescence. WB: western blot.

For the in vitro experiments (summarized in Fig. 1), we used different cell lines corresponding to dopaminergic neurons and major glial cells involved in dopaminergic degeneration: N27 dopaminergic neuronal cells, C6 astroglial cells and microglial cells (human HMC3 and mouse BV2). In addition, human kidney HEK293 cells were used as control cells. HEK293 can be used as a positive control for transfection because they have a very high transfection efficiency^32^, and they can also be used as a negative control for the effects of AngII, as HEK293 cells do not express AT1 and AT2 receptors^33^. To study the formation of α-synuclein aggregates in different cells, we performed the SynT/Sph1 aggregation model^34–36^. This model can be used to study the effects of different treatments on α-synuclein aggregation in vitro in different cell types, as cells express a certain number of aggregate-like structures termed inclusions (see Methods section for further details).

In our in vitro experiments, the inclusions were also studied using the bimolecular fluorescence complementation (BiFC) method, which enables direct visualization of protein interactions in living cells. BIFC method is particularly suitable for the measurement of inclusion size and cell-to-cell transfer of proteins. The present BiFC method was initially developed by Lázaro and Outeiro^34,37^, by tagging α-synuclein with the VC terminus of venus protein and synphilin-1 with the VN terminus of venus protein (Fig. 1b). This leads to reconstitution of the fluorescent protein Venus when two proteins, each one carrying one half of venus, are close to each other and start to interact (Fig. 1b), which leads to the formation of fluorescent inclusions that can be analyzed. See the Methods section for further details.

**Fig. 2:**
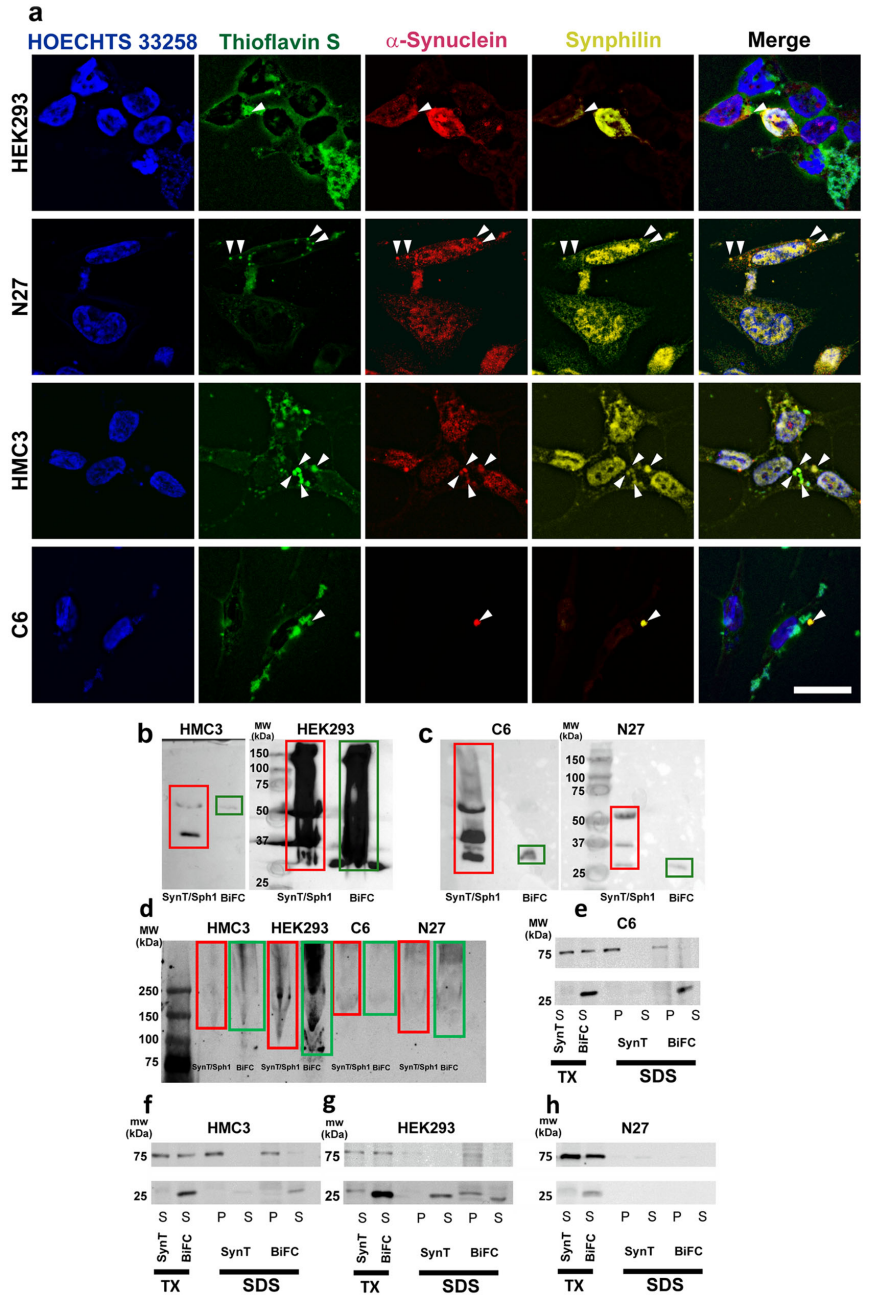
Formation of inclusions in our in vitro methods. **a** Representative pictures of HEK293, N27, HMC3, and C6 cells showing thioflavin S-positive staining for aggregates (green) α-synuclein-T (red), V5-synphilin-1 (yellow) immunoreactivity. Arrows point at possible Lewy body-like structures. Scale bar 15 µm. **b** SDD-Tricine membrane of HMC3 (left side) and HEK293 (right side) cells transfected with α-synuclein-T and V5-synphilin-1 (SynT/Sph1, red rectangle), VN-α-synuclein and α-synuclein-VC (BiFC, green rectangle). HMC3 cells show two bands of ~40 and 60 KDa when transfected with α-synuclein-T, which may be composed of α-synuclein dimers and trimers. HEK293 cells show a smear of different MW ranging from ~25 KDa to more than 150 KDa. **c** SDD-Tricine membrane of C6 (left side) and N27 (right side) cell lines transfected with SynT/Sph1 (red rectangle), VN-α-synuclein and α-synuclein-VC BiFC (green rectangle). C6 cells show several bands that may be oligomers. N27 cells show the presence of possible monomers, dimers, and trimers. **d** Native WB membrane of C6, N27, HMC3, and HEK293 cell lines transfected with SynT/Sph1 (red rectangle), VN-α-synuclein and α-synuclein-VC BiFC (green rectangle). **e**–**g** SDS-PAGE WB membranes for Triton-X and SDS solubility assays in C6, HMC3, and HEK293 cells. BiFC and SynT/Sph1 models show Triton-X soluble signals at high molecular weight. **h** SDS-PAGE WB membranes for Triton-X and SDS solubility assays in N27 cells. No signal has been observed in SDS-soluble fractions after Triton-X digestion (*N* = 4 independent experiments). TX: Triton-X. P: Pellet (insoluble) fraction. S: Soluble fraction.

As an initial test, we checked if the different cell lines used in the present study formed aggregates when transfected with α-synuclein-T and V5-synphilin-1 (SynT/Sph1 model). As the model has been mostly applied in H4 neuroglioma cell line and modifications of it in SH-SY5Y^36,38^, we assessed if the inclusions formed are indeed α-synuclein aggregates. We initially performed thioflavin S (ThS) staining combined with immunofluorescence for α-synuclein and V5 (tagged to synphilin-1) (Fig. 2a). Our results showed the presence of inclusions positive for ThS, V5 (tagged to synphilin-1) and α-synuclein in the neuronal, microglial and astroglial cells, as well as the control HEK293 cells. This confirmed the aggregate-like nature of the inclusions, and the possibility of these aggregates having a Lewy body-like structure, as LBs have been proposed to be composed of misfolded α-synuclein on the outside and synphilin-1 on the inside^39^. Additionally, to further confirm the formation of aggregates, we performed SDD-tricine western blots^40,41^. We observed the presence of oligomers in HMC3 and N27 cell lines, as the molecular weight of the monomers are 15 kDa for endogenous α-synuclein and 23 kDa for α-synuclein-T oligomer and higher molecular weight species were observed. We also observed the presence of oligomers and higher molecular weight species in HEK293 and C6 cell lines (Fig. 2b, c).

Additionally, we analyzed the presence of high molecular weight species by native non-denaturing western blots. In native conditions, our results showed that the higher molecular weight species are present in both the SynT/Sph1 model and the BiFC model, rendering both methods comparable for the assessment of inclusion size (Fig. 2d). Furthermore, western blots of samples digested in Triton-X showed that regardless of the method used, aggregates can be observed, and subsequent changes in SDS solubility are more related to the nature of the aggregates in the different cell lines than to the inclusions coming from the SynT/Sph1 model or the BiFC model (Fig. 2e–g). It was observed that in N27 cells, there was no α-synuclein signal in the SDS fractions, which points to a different nature of the inclusions in these cells in comparison with the other cell lines (Fig. 2h).

### Treatment with AngII increases the number of cells with inclusions and the average inclusion size in neuronal and microglial cells

**Fig. 3:**
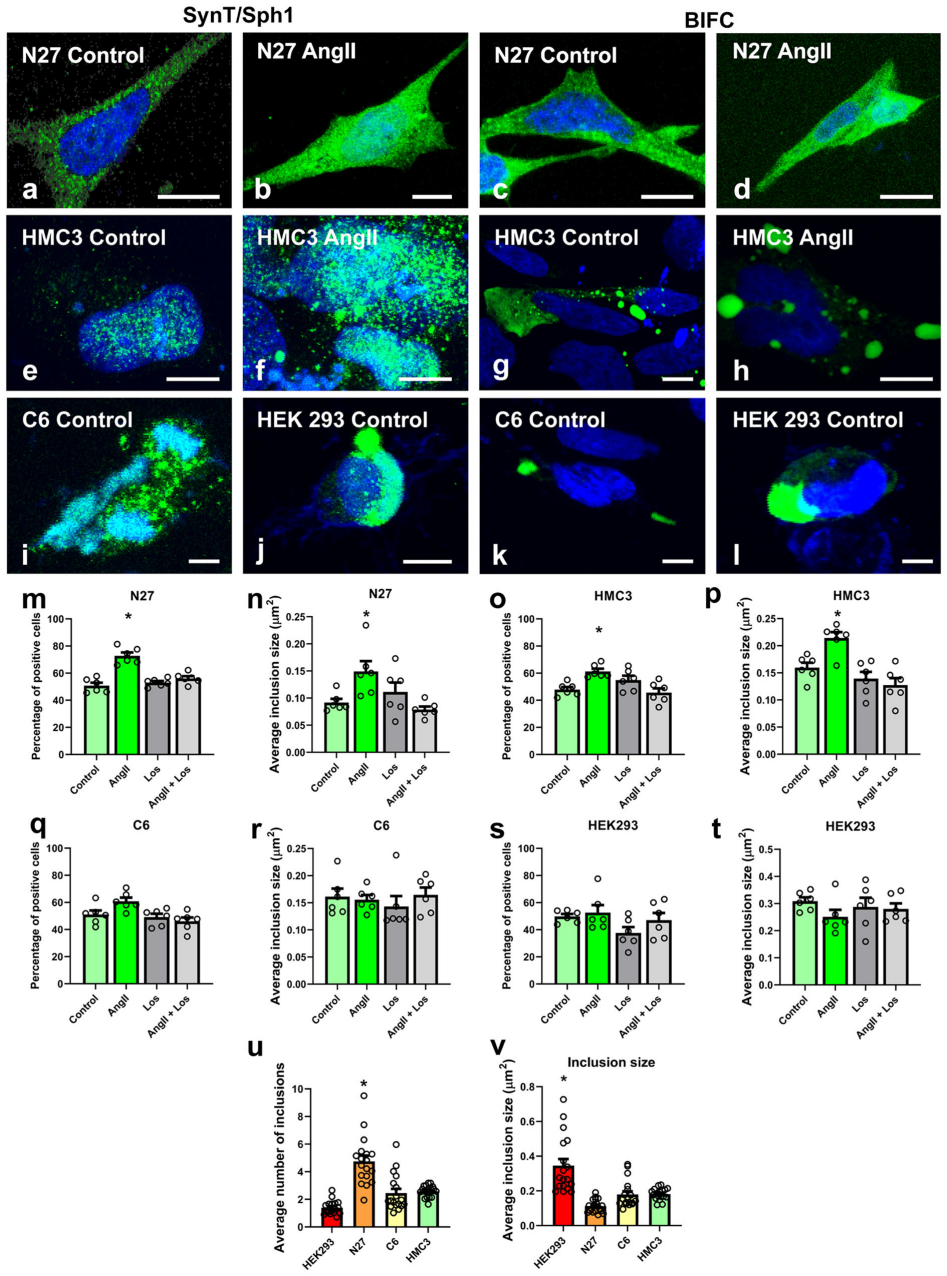
AngII increases the percentage of cells with inclusions and the inclusion size. Cells were transfected with α-synuclein-T and V5-synphilin-1 to measure number of cells with inclusions, and cells were transfected with Sph1VN/SynVC to measure the size of the inclusions by BiFC. **a**–**l** Representative confocal images of the cells analyzed. Green: α-synuclein; Blue: Hoechst 33342. **m**–**p** Treatment with AngII in N27 and HMC3 cells leads to a significant increase in the percentage of cells showing inclusions and in the average inclusion size that are reverted with treatment with losartan. **q**–**t** Treatment with AngII in C6 and HEK293 cells did not lead to significant changes in the percentage of positive cells, nor in the average inclusion size. *N* = 6 independent experiments, at least 30 pictures were analyzed for each treatment and experiment. **u** N27 cells show a significantly higher number of inclusions per cell, as observed by fluorescence microscopy using SynT/Sph1 method. **v** Average inclusion size in the different cell lines as observed by fluorescence microscopy using the Sph1VN/SynVC BiFC method. HEK293 cells show significantly bigger inclusions than N27, C6, and HMC3 cells. *N* = 18 independent experiments. Each experiment consists of approximately 30 pictures analyzed per cell group. Scale bars 14 μm (**a**–**j**, **l**), 22 μm (**k**). AngII: Angiotensin II. Los: Losartan. *p < 0.05 relative to controls. One-way ANOVA and Tukey’s multiple comparison tests. Error bars represent SEM.

After the aggregate-like structure of the inclusions was confirmed, we studied the effects that AngII/AT1 activation may have on the number and nature of the inclusions in the different cell types. We counted the number of inclusions formed with the SynT/Sph1 model, and the average inclusion size with the combination of the SynT/Sph1 model with BiFC (Fig. 3a–l and supplementary Fig. 1). In the N27 dopaminergic neuron cell line, treatment with AngII significantly increased the percentage of cells positive for inclusions, which was inhibited by treatment with the AT1 receptor blocker losartan (Fig. 3m). Furthermore, we observed a significant increase in the average inclusion size (Fig. 3n). These results suggest that AngII induces the formation of a higher number of bigger α-synuclein aggregates in dopaminergic neurons.

In the HMC3 microglial cell line, we also observed a higher number of cells showing inclusions and a higher average inclusion size after treatment with AngII, which is inhibited by treatment with the AT1 blocker losartan (Fig. 3o, p), pointing, like in N27 dopaminergic cells, to the presence of a higher number of bigger α-synuclein aggregates. In the C6 astrocyte cell line, treatment with AngII did not induce significant changes in inclusion number or size (Fig. 3q, r). This may be because astrocytes are a major source of endogenous AngII, which is already present in the culture medium, while neurons and microglia do not or practically do not release AngII to the medium (see Discussion). In the HEK293 cell line, we did not observe any significant change after treatment with AngII and/or losartan (Fig. 3s, t), which could be expected as it is known that HEK293 cells lack AT1 receptors^33^, confirming the validity of our model. In addition, the results revealed that in these cell lines, treatment with losartan alone did not induce any significant decrease in the percentage of positive cells that show α-synuclein inclusions (supplementary Fig. 1).

When we analyzed the number of inclusions formed with the SynT/Sph1 model inside the different cell types, we observed a significantly higher number of inclusions in N27 dopaminergic neurons (Fig. 3u), and that HEK293 cells have a lower number of inclusions. We also analyzed the average size of the inclusions via the BiFC method combining SphVN/SynVC, observing a significantly higher inclusion size in HEK293 cells and a smaller size in N27 cells (Fig. 3v). Together, all these results suggest the formation of a higher number of smaller aggregates in dopaminergic neurons, and a lower number of bigger aggregates in HEK293 cells, while C6 astroglia and HMC3 microglia aggregates occupied a middle position in size and number.

### Treatments with AngII and/or losartan do not change levels of α-synuclein expression, phosphorylation, or transfection in the in vitro models

**Fig. 4:**
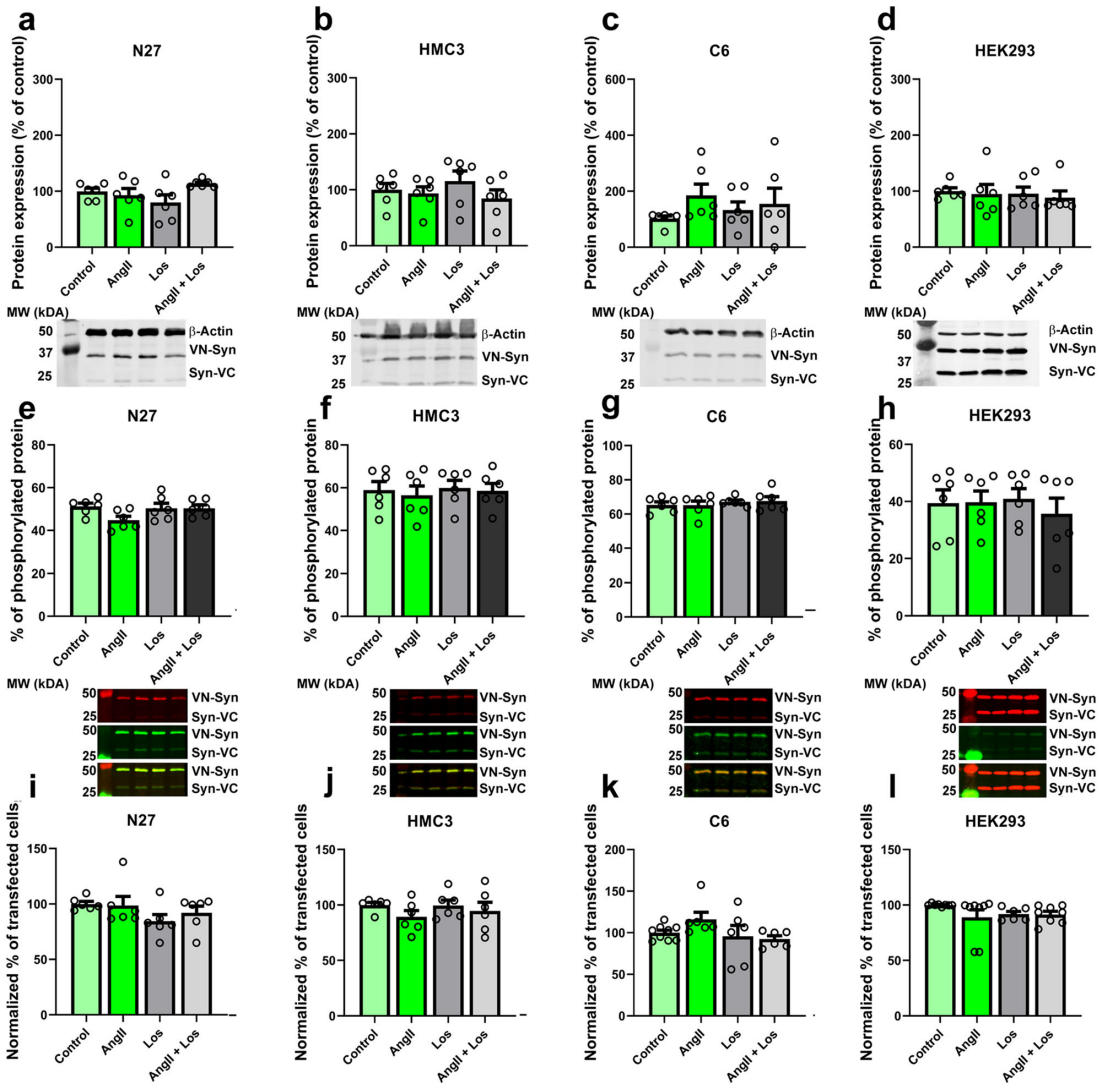
Western blot analyses show no significant changes in α-synuclein expression neither in α-synuclein phosphorylation. Cells were transfected with VNSyn/SynVC to measure both α-synuclein expression and transfection efficiency. **a**–**d** Western blot analyses of N27, HMC3, C6, and HEK293 cells with different treatments showed no significant changes due to the different treatments. *N* = 6. **e**–**h** Western blot analyses of N27, HMC3, C6, and HEK293 cells showed no differences in α-synuclein phosphorylation levels due to the different treatments. Green = Phosphorylated α-synuclein. Red = not phosphorylated α-synuclein. *N* = 6. **i**–**l** Flow cytometry analyses of the different cell treatments do not show any differences in α-synuclein transfection for VNSyn and SynVC plasmids. *N* = 6. AngII Angiotensin II, Los Losartan. One-way ANOVA and Tukey’s multiple comparison tests. Error bars represent SEM.

To know whether the changes observed in the number of inclusions or the size of the inclusions after treatment with AngII or losartan may be due to changes in α-synuclein expression, we performed SDS-PAGE western blots of cells. In transfected dopaminergic neurons, astroglial, and microglial cells, we did not observe significant changes in α-synuclein expression related to AngII or losartan treatments (Fig. 4a–d). Furthermore, as α-synuclein phosphorylation at the serine 129 may change the ability of α-synuclein to form aggregates or the internalization of α-synuclein^42^, we performed two additional experiments. First, we measured by fluorescent western blotting the phosphorylation levels of α-synuclein after the different treatments. Our results did not show any changes in α-synuclein phosphorylation levels (Fig. 4e–h). Second, we measured changes in the number of transfected cells using flow cytometry and BiFC, as transfected cells will be those that express and internalize both α-synuclein constructs. We did not observe any changes in the number of transfected cells, which indicates that both VN- α-synuclein and α-synuclein-VC are transfected and expressed normally by the cells after the different treatments (Fig. 4i–l; supplementary Figs. 2–4); supplementary Tables 1, 2). Therefore, the changes mentioned above in the number and the size of the aggregates induced by AngII or the AT1 receptor blocker losartan (Fig. 3) are not due to changes in α-synuclein expression/transfection rates, neither due to changes in phosphorylation nor transfection levels.

### NADPH-oxidase activation and intracellular calcium raising mediate AngII/AT1-induced increase in α-synuclein aggregation

**Fig. 5:**
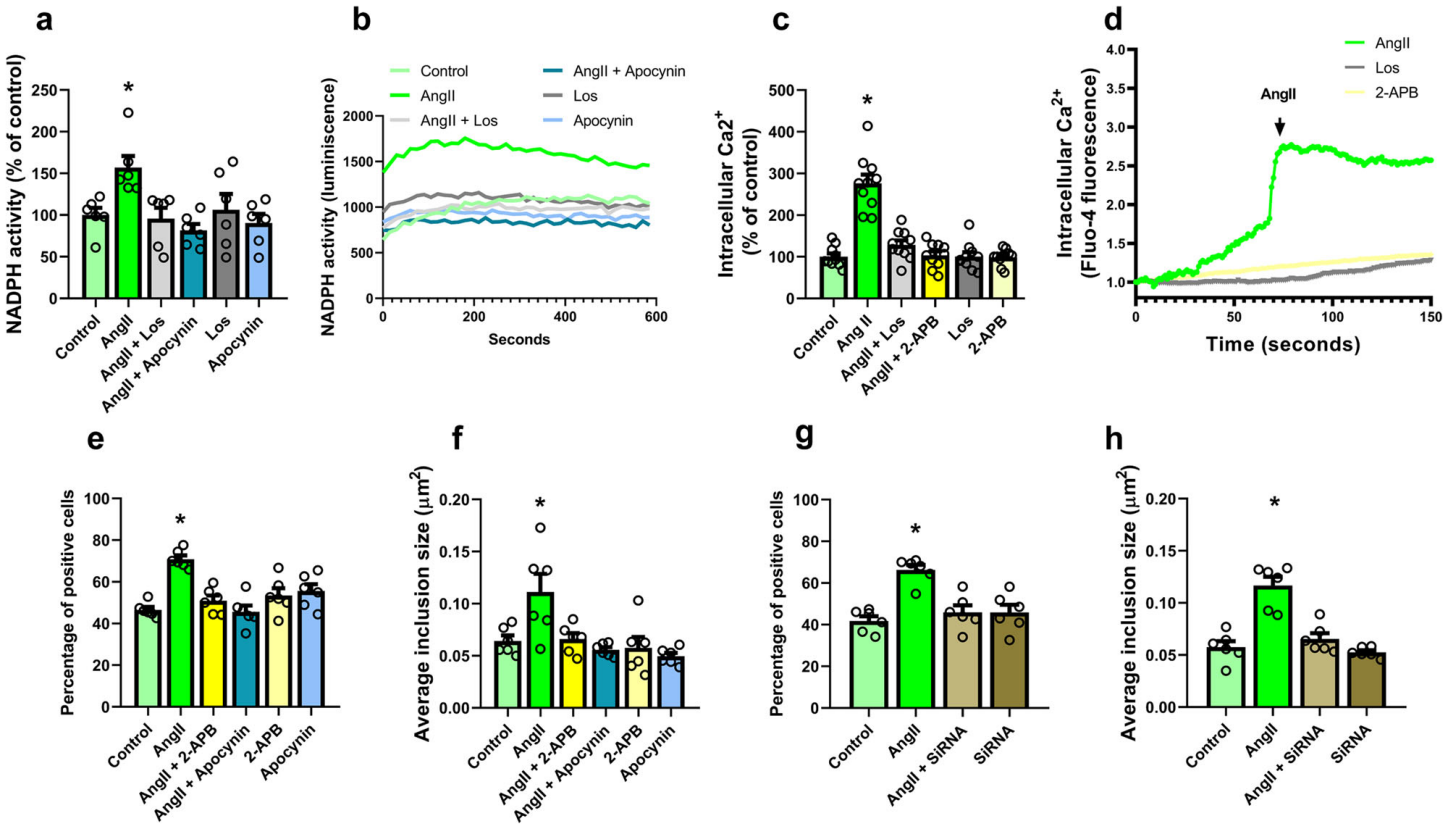
NADPH-oxidase activation and intracellular calcium raising mediate AngII/AT1-induced increase in α-synuclein aggregation. **a**, **b** Treatment with losartan or apocynin blocks the effect of AngII on NADPH-oxidase activation. **c**, **d** Treatment with losartan or 2-APB blocks the effect of AngII on intracellular calcium release. For calcium release measurements, *N* = 10 pictures were used; individual regions of interest were analyzed for each picture. **e**, **f** Treatment with the calcium influx inhibitor 2-APB or with the NADPH-oxidase apocynin blocks the effects of AngII on the percentage of cells showing inclusions and the average inclusion size in N27 cells. **g**, **h** Partial blockade of the expression of the NAPDH-oxidase gp91 subunit with short interference RNA blocks the effects of AngII on the percentage of cells showing inclusions and the average inclusion size in N27 cells. *N* = 6 independent experiments, between 20 and 34 pictures were analyzed for each treatment and experiment. AngII: Angiotensin II. Los: Losartan *p < 0.05 relative to controls. One-way ANOVA and Tukey’s multiple comparison test. Error bars represent SEM.

Previous studies in different cell types have shown that major effects of AngII/AT1 are mediated by NADPH-oxidase complex activation, which leads to an increase in intracellular levels of superoxide, and intracellular calcium raising. We confirmed that this was also occurring in our cell model. Consistent with previous studies, we observed an increase in NADPH-oxidase activity in N27 dopaminergic cells after treatment with AngII, which was blocked both by treatment with the AT1 blocker losartan and the NADPH-oxidase inhibitor apocynin (Fig. 5a, b). In addition, we confirmed that AngII increases intracellular calcium in N27 cells and that this increase is blocked both by treatment with the AT1 blocker losartan and the calcium channel inhibitor 2-APB (Fig. 5c, d).

Treatment with the calcium channel inhibitor 2-APB or with the NADPH-oxidase inhibitor apocynin blocked the effects of AngII on the percentage of cells showing inclusions and the average inclusion size in N27 dopaminergic neurons (Fig. 5e, f; supplementary Fig. 1). Given that the current NADPH-oxidase inhibitors, including apocynin, are not totally specific, we confirmed the involvement of NADPH-activation in the above-mentioned effects of AngII on α-synuclein inclusions by performing partial blockage of the expression of the gp91 subunit of the NADPH-oxidase with short interference RNA, which also inhibited the effects of AngII on the percentage of cells showing inclusions and the average inclusion size in N27 dopaminergic neurons (Fig. 5g, h; supplementary Fig. 1). All these results reveal that the increase in NADPH-oxidase-derived superoxide and the intracellular calcium levels induced by activation of AT1 receptors are responsible for the above-mentioned effects of AngII/AT1 on α-synuclein aggregation.

### Validation of an in vivo model: α-synuclein aggregation in MPTP-treated mice

**Fig. 6:**
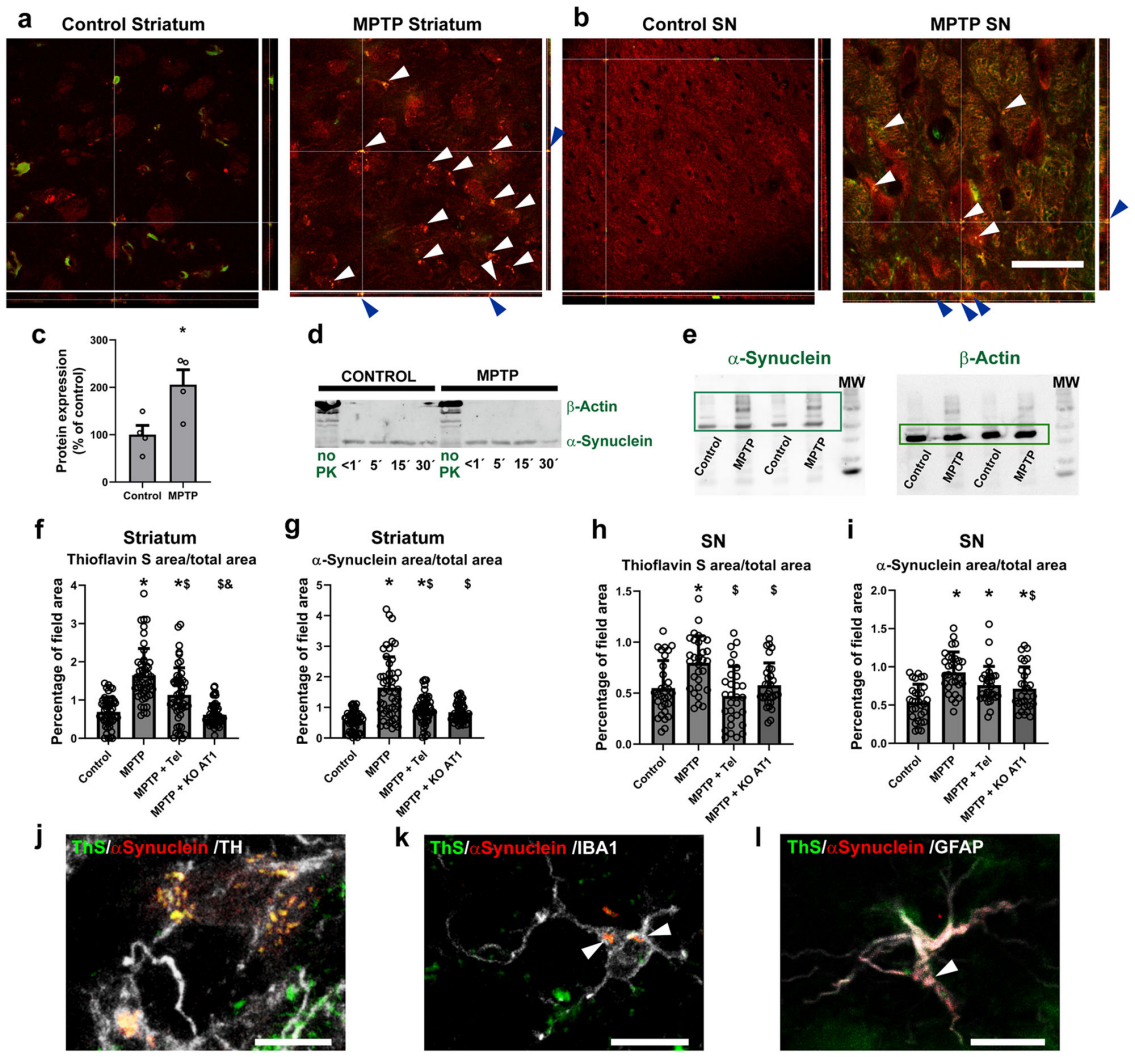
Striatum and substantia nigra of control mice and MPTP-treated mice. **a**, **b** Treatment with MPTP induced the formation of inclusions in the striatum (**a**) and nigra (**b**), as observed in confocal pictures with projections in all three dimensions, where white and blue arrows point to the inclusions. Scale bar: 50 µm; Red: α-synuclein. Green: ThS. **c** Treatment with MPTP led to higher levels of α-synuclein oligomer phosphorylation at the Serine 129 as measured by western blot. **d** Treatment with MPTP did not change the resiliency of aggregated α-synuclein oligomers to pK digestion. **e** Treatment with MPTP induced the formation of higher molecular weight species in mouse substantia nigra as observed by SDD-tricine western blot. **f**, **i** Treatment with MPTP led to significant increase in thioflavin S-positive staining (**f**, **h**) and in α-synuclein expression (**g**, **i**) in the striatum (**f**, **g**) and substantia nigra (**h**, **i**), which were inhibited by treatment with the AT1 blocker telmisartan or the absence of AT1 receptors (AT1 KO mice). *N* = 50 (**f**, **h**) or 30 (**i**) pictures analyzed. **j**–**l** Double immunofluorescence and ThS staining of mouse tissue showing the presence of α-synuclein aggregates in nigral dopaminergic neurons (**j**), microglia (**k**), and astrocytes (**l**). Scale bar: 10 µm. **j**–**l** Tel: Telmisartan. MPTP + KO AT1: MPTP-treated AT1 KO mice. Red: α-synuclein. White: TH (**j**), IBA-1 (**k**), GFAP (**l**). Green: ThS aggregates. *p < 0.05 relative to control. ^$^*p* < 0.05 relative to MPTP. ^&^*p* < 0.05 relative to MPTP + Telmisartan. Unpaired two-tailed *t* test (**c**). Two one-way ANOVA and Tukey’s multiple comparison test (**j**–**l**). Error bars represent SEM.

After we observed the changes that RAS modulation causes in α-synuclein aggregation in different cell models, we decided to confirm these changes in vivo in a murine model. As commented above, controversial results were reported on the presence of α-synuclein aggregates in the MPTP mouse model of Parkinsonism. Therefore, we initially studied if our MPTP-treated mice showed the presence of α-synuclein aggregates using confocal microscopy. In our confocal images, we clearly observed the presence of aggregates positive for Thioflavin S staining and immunoreactive for α-synuclein, as shown in tridimensional projections (Fig. 6a, b; supplementary movies 1–4). To further confirm the validity of the model, we performed additional experiments in homogenates from mouse substantia nigra. As we mention below, aggregated α-synuclein is mostly phosphorylated at the serine 129. So, to further confirm our in vivo results, we measured by western blotting the phosphorylation levels of α-synuclein at the serine 129 in homogenates from mouse substantia nigra. We observed an increase in phosphorylation in MPTP-treated mice (Fig. 6c; Supplementary Fig. 8j). Additionally, we performed a series of digestions of the mouse substantia nigra homogenates with proteinase K (pK) as more tightly packed aggregates are more resilient to pK digestion. We observed no clear changes in resiliency to pK digestion between control and MPTP-treated mice (Fig. 6d) independently of the total amount of α-synuclein. Finally, similarly to our in vitro experiments, we performed SDD-tricine western blotting, where we observed a stronger signal in high molecular weight species in MPTP-treated mice, which points to higher quantities of aggregates (Fig. 6e). In summary, our results show that, even though the aggregates themselves are not more tightly packed, there is a significantly higher number of pathological α-synuclein aggregates in the MPTP mouse model of Parkinsonism.

### Modulation of RAS leads to changes in α-synuclein aggregation in MPTP-treated mice

We studied changes in the aggregates by fluorescent microscopy analysis in C57BL/6 J mice treated with MPTP alone, mice treated with MPTP and the AT1 blocker telmisartan, and MPTP-treated mice that do not express AT1 receptors (AT1 KO). As in the present study, losartan is usually chosen as an effective AT1 blocker for in vitro experiments. However, losartan is not effective at crossing the blood–brain barrier. Telmisartan is most appropriate for in vivo models, as it is one of the most effective AT1 blockers at crossing the blood–brain barrier, and low doses not affecting blood pressure are able to induce effects on brain RAS in vivo^10,43^.

We analyzed at least 30 brain sections throughout the striatum and the substantia nigra from 6 different mice by group. Both in the striatum and nigra, the results obtained from image analysis showed a significant increase in ThS-stained aggregates after subacute treatment with MPTP, which was inhibited by treatment with the AT1 receptor blocker telmisartan and in AT1 KO mice. Furthermore, we observed a significant increase in the expression of α-synuclein after subacute treatment with MPTP, which was inhibited by treatment with the AT1 receptor blocker telmisartan and in AT1 KO animals (Fig. 6f–i). These results confirm, under our experimental conditions and analytical methods, the formation of aggregates in mouse nigrostriatal system, which is combined with a significant increase in α-synuclein expression after subacute MPTP treatment, and that AngII, via AT1 receptors, plays a major role in this process. Furthermore, consistent with our in vitro observations, we observed the presence of aggregates mainly in dopaminergic neurons and terminals but also in astrocytes and microglia of MPTP-treated mice. We performed double immunofluorescence for the corresponding cell type marker combined with the aggregate marker ThS. Our analyses showed a significant ThS staining and α-synuclein expression colocalizing with remaining TH-positive neurons and striatal terminals (Fig. 6j). Furthermore, ThS-stained aggregates and α-synuclein immunoreactivity was also found colocalizing with microglial marker IBA-1 (Fig. 6k) and the astroglial marker GFAP (Fig. 6l).

### Release and uptake of α-synuclein are different between cell types

**Fig. 7:**
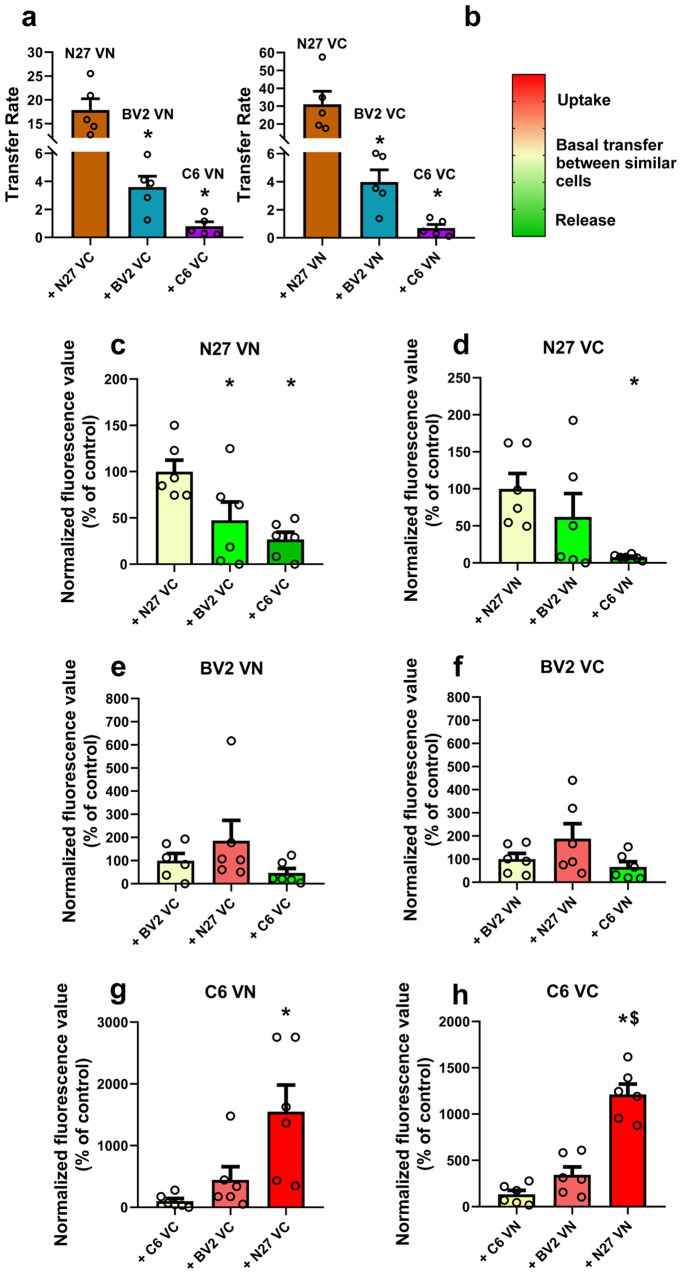
α-synuclein differential cell-to-cell transfer. Cells were transfected either with VNSyn or SynVC to measure α-synuclein transmission by BiFC. **a** N27 cells show significantly higher α-synuclein transfer rate than BV2 cells and C6 cells. **b** Color code for **c**–**h**: light yellow indicates the rate of transfer between cells of the same type shown in **a**, red color indicates uptake of α-synuclein, while green color indicates release of α-synuclein by the cells indicated on top of each histogram when mixed with the cells indicated under each column of the histogram. **c**, **d** N27 cells manifest a significant increase in their release rate of α-synuclein when co-cultured with C6 cells and a significant increase in the release rate of VN-α-synuclein when co-cultured with BV2 cells expressing α-synuclein-VC. **e**, **f** BV2 cells manifest mild, not significant trends in their α-synuclein transfer when co-cultured with other cell types: uptake with N27 and release with C6. **g**, **h** C6 cells show a significant increase in their α-synuclein uptake when co-cultured with N27 cells. *N* = 6 independent (10,000 events analyzed) flow cytometry measurements. *p < 0.05 relative to controls (yellow values). One-way ANOVA and Dunnet’s multiple comparison test. Error bars represent SEM.

To study possible α-synuclein transmission between different cell types (particularly dopaminergic neurons, astroglial cells, and microglial cells), we performed cell co-cultures (via transwell) and counted fluorescent cells using the BiFC method and flow cytometry. Using a transwell system we analyzed each cell type separately. We initially assessed the fluorescence levels of cells belonging to the same cell type expressing either VN or VC fragments and established them as control values. We then proceeded to assess by flow cytometry the fluorescence levels of co-cultures of different cell types expressing either VN or VC fragments (Fig. 7 and supplementary Figs. 5–7; supplementary Table 3).

For this set of experiments, we used microglial BV2 cells as HMC3 microglial cells were less suitable. BV2 cells show good transfection rates for single plasmids and good survival in co-cultures. Additionally, during cell-to-cell transfer, as BV2 cells are smaller than HMC3 cells, they are more easily detected by the flow cytometer. However, BV2 cells were less suitable than HCM3 for the above-described aggregation experiments because BV2 cells have very low transfection rates for the double transfection of the SynT/Sph1 model leading to not enough transfected cells to properly quantify different treatments (see Methods section for details).

The results showed a high level of transfer of α-synuclein between N27 dopaminergic neurons, which was significantly higher than the transfer of α-synuclein between BV2/BV2 and C6/C6 (Fig. 7a). Transfer rates between different types of cells are shown using a color code (Fig. 7b), where a stronger red color indicates stronger uptake, light yellow indicates the rate of transfer between cells of the same type (shown in Fig. 7a), and a stronger green color indicates stronger release by the cells indicated on top of each histogram when mixed with the cells indicated under each column of the histogram (Fig. 7c–h). Our results showed a higher release of α-synuclein from N27 dopaminergic cells when mixed with BV2 and C6 cells (Fig. 7c, d; supplementary movie 5). Similarly, we observed an increase in fluorescence levels of BV2 (non-significant) and C6 cells when mixed with N27 cells, pointing to a higher uptake of α-synuclein by these glial cell types in the presence of N27 dopaminergic neurons (Fig. 7e–h). Our results strengthen the hypothesis of a strong transfer of α-synuclein from neurons to astrocytes and microglia but not significant transfer from glia to neurons. Furthermore, we observed a trend (not significant in the present experiments) to transfer α-synuclein from BV2 to C6 cells, suggesting the possibility of α-synuclein transfer from microglia to astrocytes.

### Effects of AngII on the release and uptake of α-synuclein between different cell lines

**Fig. 8:**
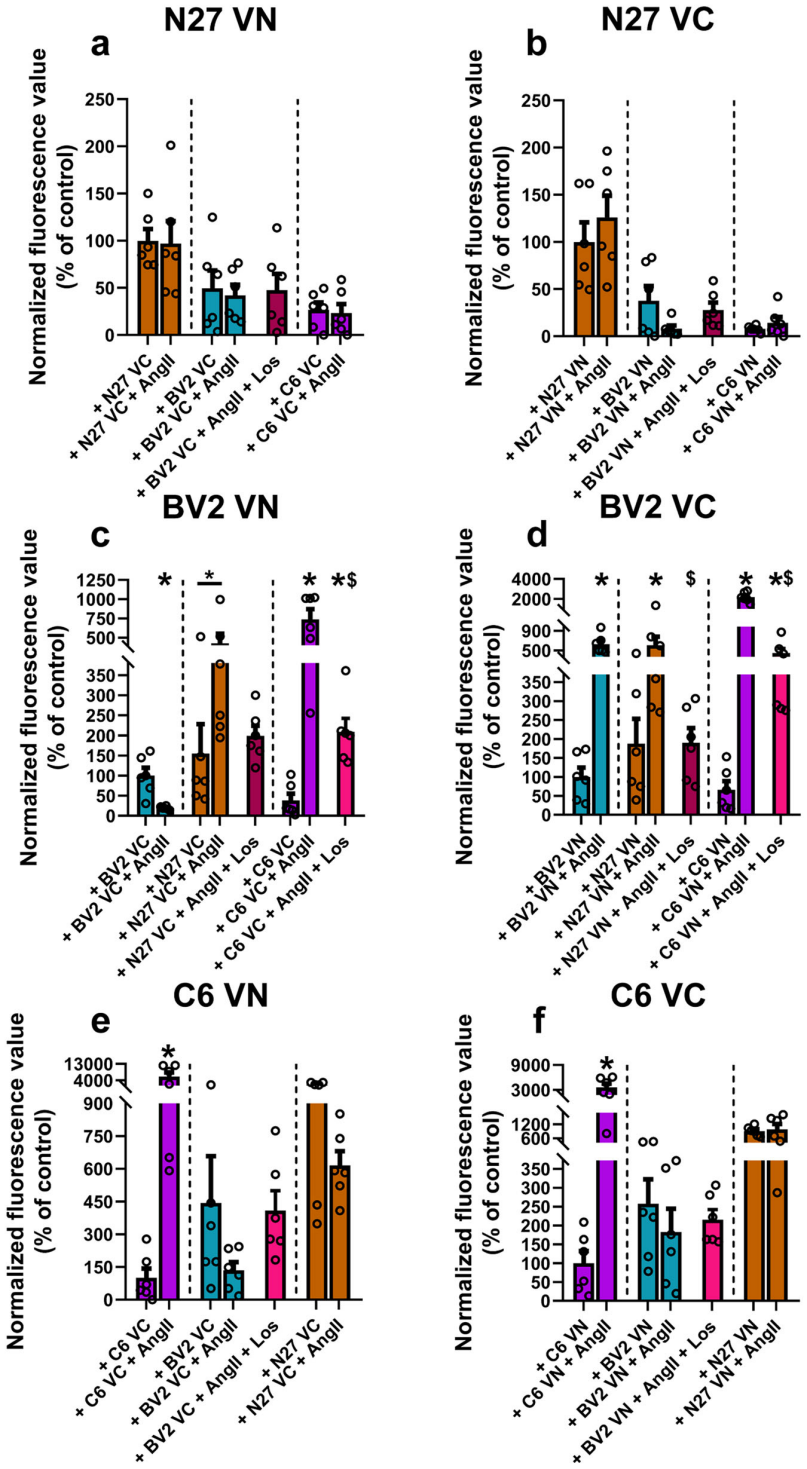
Effect of treatment with AngII on cell-to-cell transfer. Cells were transfected either with VNSyn or SynVC to measure α-synuclein transmission by BiFC. **a**, **b** Treatment with AngII did not lead to any significant changes in protein release or uptake by N27 cells. **c**, **d** Treatment with AngII led to a significant increase in protein uptake by BV2 cells when co-cultured with N27 or C6 cells, which can be inhibited by treatment with losartan. Amongst BV2 cells, treatment with AngII leads to a significant increase in the release of VN-α-synuclein. A significant decrease in the percentage of fluorescent BV2 cells expressing VN-α-synuclein can be observed together with a significant increase in the percentage of fluorescent BV2 cells expressing α-synuclein-VC. **e**, **f** Treatment with AngII leads to a higher α-synuclein transfer between C6 cells, showing a significant increase in the percentage of fluorescent C6 cells expressing α-synuclein-VC; unlike with BV2 cells where the transfer happens in one direction, in C6 cells it happens in both directions. *N* = 6 independent (10,000 events analyzed) measurements. *p < 0.05 relative to untreated cells. ^$^*p* < 0.05 relative to AngII treated cells. Unpaired two-tailed *t* test or Mann–Whitney test for pairwise comparisons or Brown-Forsythe and Welch ANOVA for multiple comparisons. Error bars represent SEM.

After assessing the differences in the release and uptake of α-synuclein between the different cell lines, we studied the effects of the addition of AngII. The rationale of these experiments was similar to that used to assess the differences in release and uptake, i.e., we employed the BiFC technique and cells co-cultured via the transwell system (Fig. 8, supplementary Fig. 5–7). Although treatment with AngII induces an increase in α-synuclein aggregation (see above), AngII did not lead to any significant changes in protein release or uptake by N27 cells relative to their basal rate (Fig. 8a, b). However, treatment with AngII led to higher α-synuclein internalization by BV2 cells when mixed with C6 cells or N27 cells, indicating that AngII increases the ability of microglial cells to capture α-synuclein, and these changes are blocked by treatment with losartan, showing that the effects are mediated by activation of the AngII/AT1 pathway (Fig. 8c, d). Regarding C6 astroglial cells, significant changes are observed when C6 cells are mixed with C6 cells; leading to an increase in the percentage of positive C6 cells, which suggests an increase in α-synuclein transfer among C6 cells (Fig. 8e, f). C6 astroglial cells show a high level of α-synuclein uptake from N27 neurons in the presence or absence of exogenous AngII, possibly related to C6-derived AngII release (Fig. 7g, h).

## Discussion

α-Synuclein is mainly expressed in the brain, particularly in neurons and, at low levels, in glial cells, and at low levels in several peripheral organs. Although α-synuclein is expressed mainly in neurons, α-synuclein aggregates were observed in astrocytes in several neurodegenerative diseases^44^, and microglial cells have been suggested to uptake and degrade α-synuclein released by neurons^5^. It is commonly considered that glial cells accumulate α-synuclein aggregates by up-taking extracellular α-synuclein secreted by neurons to regulate α-synuclein homeostasis in the brain^45,46^. In the present study, we showed the capacity for the formation of aggregates/inclusions not only by N27 dopaminergic neurons, but also by astrocytes, microglia, and HEK293 cells. The size and number of inclusions varied depending on the cell type, with a higher number of smaller aggregates in N27 dopaminergic neurons, and a lower number of bigger aggregates in HEK293 cells. In the present study, only the levels of exogenous α-synuclein were measured. Endogenous levels of α-synuclein were very low or even not detectable in western blot membranes (supplementary Fig. 8). In the membranes where it can be detected, the levels are consistently below 4% of total α-synuclein. In fact, previous literature also showed very low levels of endogenous alpha-synuclein in N27 dopaminergic cells, which was basically ignored^47^. We therefore considered the effects of endogenous α-synuclein negligible in relation to the total levels.

We also observed that activation of the AngII/AT1 axis affects α-synuclein aggregation and cell-to-cell α-synuclein transmission. Our in vitro results show that activation of AT1 receptors by AngII leads to an increase in α-synuclein aggregation in neurons and microglial cells. As expected, no effects were observed in HEK293 cells, which lack AT1 receptors. However, we did not detect any significant increase in α-synuclein aggregation in C6 astrocytes after treatment with AngII. A possible explanation is that astrocytes already produce angiotensinogen/AngII, being the main brain source of AngII^16,17^. In cultures of primary astrocytes or C6 astroglial cells, we have previously shown that treatment with exogenous AngII induced a decrease in levels of astroglial angiotensinogen and down-regulation of AT1 receptors, which was inhibited by simultaneous treatment with the AT1 blocker candesartan, suggesting a feedback mechanism of regulation of endogenous angiotensinogen production by high levels of extracellular AngII^16^. This may counteract and attenuate the effects of the treatment of C6 cultures with additional exogenous AngII in the present experiments.

Several mechanisms may be involved in the increase in α-synuclein aggregation by AngII/AT1 overactivity. Increased expression or phosphorylation of α-synuclein may affect aggregation^42,48^. In a healthy brain, most α-synuclein is unphosphorylated, while aggregated α-synuclein in LB is mostly phosphorylated at Ser129. However, we observed that the changes observed in the different cell lines after treatment with AngII are not due to AngII-induced changes in α-synuclein expression, which in the in vitro models appears more related to the transfection efficiency, nor change in α-synuclein phosphorylation. Interestingly, previous studies have observed that oxidative stress, particularly that induced by NADPH-oxidase-derived superoxide, promotes α-synuclein aggregation into oligomers^49–51^. In addition, the increase in intracellular free calcium also promotes α-synuclein aggregation^52^, and both calcium and oxidative stress cooperate to synergistically enhance α-synuclein aggregation^50^, so that calcium binding alone induces non-stable aggregates, whereas calcium together with oxidative stress induces larger and more stable aggregates^52,53^. Conversely, α-synuclein aggregation raised calcium levels and oxidative stress^54,55^, which may lead to a vicious cycle. It is well-known that a major mechanism induced by activation of the AngII/AT1 axis is the increase in the generation of superoxide derived from NADPH-oxidase complex activation, which produces high levels of superoxide in cells responsible for the inflammatory response, such as microglial cells, and lower levels of superoxide in tissue-resident cells such as neurons^56^. In addition, the interaction between NADPH-oxidase-derived superoxide and mitochondria, via mitochondrial ATP-sensitive potassium channels (mitoKATP), induces a further increase in cell ROS levels, as shown in several cell types, including dopaminergic neurons^19,20^. AngII/AT1 activity also induces a persistent cytosolic calcium increase in neurons and glial cells^21–23^. A synergistic effect of superoxide and calcium rise may explain the Ang/AT1-induced increase in α-synuclein aggregation observed in the present study. In the present experiments, we showed that the activation of NADPH-oxidase and changes in calcium homeostasis induced by AngII/AT1 activation mediate the increase in α-synuclein aggregation in dopaminergic neurons, which was inhibited by NADPH-oxidase and calcium influx inhibitors. It is also interesting to note that, in our experiments, treatment with losartan alone did not lead to significant changes in α-synuclein aggregation in dopaminergic neurons and glial cells. Previous studies have proposed that losartan alone may interact with amyloid proteins such as amyloid-β and α-synuclein through mechanisms independent from the AngII/AT1 pathway^57,58^. In our models, we did not observe these proposed interactions, suggesting that losartan effects are dependent on its inhibition of the AngII/AT1 pathway.

As stated above, there was some controversy on the presence of α-synuclein aggregates in the MPTP mouse model, which is probably related to the experimental conditions, particularly the dose, time-course of the MPTP administration and the method used to detect the α-synuclein aggregates^27,29,31^. In the subacute MPTP model used in the present study, we clearly observed a significant increase in the expression of α-synuclein and aggregates in the nigra and striatum, which were observed particularly in dopaminergic neurons and terminals, but also in astrocytes and microglia. Consistent with the in vitro observations, AngII/AT1 activation is involved in the increase in α-synuclein expression and aggregation in MPTP-treated mice, as they significantly decreased in mice treated with the AT1 blocker telmisartan and in AT1 KO mice. This is consistent with the well-known increase in oxidative stress and microglial neuroinflammatory response that mediates the MPTP neurotoxicity^8,59^, and particularly with previous studies from our group and others showing that AT1 receptor blockers inhibit the oxidative stress and neuroinflammation in the MPTP mouse model, leading to a reduction in dopaminergic neuron degeneration^8,20^. This is further supported by the observation that generation of oxidative stress in MPP^(+)^-treated dopaminergic neurons occurs as a NADPH-oxidase-dependent two-wave cascade that involves both NADPH-oxidase- and mitochondrial-derived superoxide and that this is inhibited by treatment with AT1 receptor blockers^19,20,59,60^. Consistent with this, it was also observed that MPP^(+)^ can act directly on astrocytes enhancing angiotensinogen/AngII production^16^. Both oxidative stress (see above) and neuroinflammation^61^ promote α-synuclein expression and aggregation, and both are induced by MPTP treatment and enhanced by AT1 activation^8,20^, which supports the results observed in the present experiments in mice.

Several previous studies have observed that the MPTP-induced murine inclusions appeared different from the classical nigral Lewy bodies, and closely resembled human cortical inclusions and the dense, granular core synuclein-immunoreactive granules of classical nigral LBs, suggesting that MPTP-induced inclusions may reflect the initial stages in the formation of nigral Lewy bodies^62–64^. It is interesting to consider that most literature pertaining α-synuclein aggregate structure in mice is more than one decade older than the most recent studies on α-synuclein aggregate structure in humans. This implies that new techniques that allowed for a clearer determination of human Lewy body structures, like cryo-electron microscopy or STED microscopy, have still not been used in the MPTP mice model^65–67^. These recent studies in humans show the presence of crowded organelles and lipid membranes in between α-synuclein filaments, with LBs harboring high quantities of lipids^65,66^. These observations may point to closer similarities between α-synuclein inclusions in MPTP-treated mice and human Lewy bodies than what was previously thought. In future studies, a detailed characterization of the MPTP-induced inclusions using the above-mentioned new methodologies would be interesting for a better knowledge of the differences between MPTP-induced inclusions and human nigral LBs. In any case, the present results show that the AT1 blockers significantly decrease the presence of these possibly initial forms of LB pathology in the MPTP mouse model.

In the present study, we performed a detailed analysis of α-synuclein transmission between dopaminergic neurons, astrocytes, and microglia using a transwell model and the BiFC method in the absence and presence of AngII. The results showed a high level of transfer of α-synuclein between N27 dopaminergic neurons, which is significantly higher than the transfer of α-synuclein between BV2/BV2, and C6/C6. We also observed a high release of α-synuclein from N27 dopaminergic cells when mixed with BV2 and C6 cells. The present results are consistent with the hypothesis of a strong transmission of α-synuclein from neurons to astrocytes and microglia and less efficient transmission from glia to neurons.

Furthermore, we observed that treatment of co-cultures with AngII induced changes in cell-to-cell transmission of α-synuclein. Interestingly, although AngII induced an increase in the size and number of aggregates in N27 dopaminergic neurons, treatment with AngII did not lead to significant changes in the rate of transfer of α-synuclein from N27 neurons to glial cells, which may lead to a further increase in accumulation of aggregates in dopaminergic neurons after activation of the AngII/AT1 axis. However, AngII led to a higher α-synuclein uptake by BV2 microglial cells when mixed with C6 cells or N27 dopaminergic neurons, suggesting that AngII increases the ability of microglial cells to capture α-synuclein. Furthermore, when AngII was added to the culture, the uptake of α-synuclein by C6 astroglial cells showed a tendency to decrease in the presence of BV2 cells (which showed a significant AngII-induced increase in the uptake from astrocytes). Furthermore, AngII induced a significant increase in the transfer of α-synuclein among C6 astroglial cells. The mechanisms responsible for the effects remain to be totally clarified. Previous studies have shown that oxidative stress, and particularly the increase in NADPH-derived superoxide enhanced cell-to-cell α-synuclein transmission^51,68^ and activation of the NADPH-oxidase complex is a major mechanism involved in AngII/AT1 effects (see above). In addition, AngII/AT1 activation increases the microglial proinflammatory response^25,26,69^, which is consistent with the marked AngII-induced increase in microglial uptake of α-synuclein observed in the present experiments. Clarification of the molecular mechanisms involved in α-synuclein cell-to-cell transmission is essential to develop effective therapies for PD and other synucleinopathies. A major point is to clarify if glial cells, particularly astrocytes, promote the removal of α-synuclein or may contribute to its propagation to neurons. The present results support the scavenger function, at least in our model. It has also been suggested that astrocytes and microglia may initially uptake and degrade α-synuclein aggregates, but may be finally unable to achieve total elimination, leading to intracellular accumulation and transferring them to other cells^5,70^. Furthermore, the incorporation of α-synuclein species by astrocytes and microglia may promote neuroinflammatory responses and progression of the disease^7,71,72^.

Cell-to-cell transfer of α-synuclein can happen through several proposed mechanisms. These include passive diffusion^73^, transport through carrier proteins^74^, exo- and endocytosis^75^, and exosomes or tunneling nanotubes^76,77^. In our experimental design, the transwell system membrane has a pore diameter of 400 nm and a nominal width of 10 μm, keeping more than 200 μm of separation between the transwell membrane and the bottom of the cell plate. Transport by tunneling nanotubes is not probable due to the distance between cells cultured in the transwell membrane and at the bottom of the cell plate well. The transwell membrane may also limit transport by vesicles of diameters above 400 nm^78^. Regarding exosomes (which have an average diameter of 100 nm), it has been reported that transwells may reduce their diffusion^79^. Thus, the main remaining mechanisms of α-synuclein cell-to-cell transfer are passive diffusion, transfer through carrier proteins, and exo- and endocytosis. In this model, cell-to-cell transfer of α-synuclein can happen for both oligomeric and monomeric forms of α-synuclein^80,81^.

In PD, several major processes are known to be involved in dopaminergic degeneration, particularly oxidative stress, neuroinflammation, and accumulation of α-synuclein aggregates. However, the reason for initiating these processes and their order of appearance remain to be clarified. It is now debated the usual view of α-synuclein aggregation and Lewy body pathology (LBP) as the initial event, and it was suggested the possibility that earlier cellular and molecular changes occur in the most vulnerable dopaminergic neurons^82,83^. Before LBP, early neurochemical changes such as a decrease in tyrosine hydroxylase^84^, oxidative damage^85^, and neuroinflammation^86^ have been reported. Previous studies in different tissues, including the nigrostriatal tissue, have revealed counterregulatory interactions between dopamine and the AngII/AT1 axis^87,88^, so that an initial decrease in dopamine levels due to an early alteration in the dopaminergic function^84^ may increase the activity of the AngII/AT1 axis, which in turn induces a compensatory increase in dopamine levels through a number of mechanisms^16,89–92^. However, AngII/AT1 overactivity may simultaneously promote oxidative stress and neuroinflammation by enhancing NADPH-oxidase activity and the increase in levels of intracellular free calcium, as described above. Interestingly, the present study shows that AngII/AT1 activation may also promote α-synuclein expression, aggregation, and cell-to-cell transmission. Other factors, including aging^25,91^, peripheral inflammation, or metabolic syndrome^93^, may also induce overactivation of the nigrostriatal AngII/AT1 axis, via agonistic AT1 receptor autoantibodies^93,94^ or other mechanisms, which particularly affects dopaminergic neurons of the nigral ventral tier with high levels of AT1 receptor expression^13^.

As far as we know, there are no studies on the possible effects of AngII/AT1 activation on α-synuclein aggregation and transmission in peripheral tissues. Among peripheral tissues, the gastrointestinal tract (GIT) is particularly interesting for PD and α-synuclein pathology in the context of the gut-brain axis. Interestingly, it is known that there are both a gut RAS^95^ and a gut dopaminergic system^96,97^ that regulate important GIT functions. In several recent studies, we have studied the role of RAS in brain-gut interactions in PD models^98^, and interactions between the gut RAS and gut dopaminergic system in models of higher risk factors for PD such as aging^99^ or menopause^100^. However, a possible role of the dysregulation of gut RAS/dopamine interactions in gut α-synuclein pathology remains to be clarified in future studies.

In conclusion, AngII/AT1 overactivity and brain RAS dysregulation appear involved in major processes related to PD progression, such as oxidative stress, neuroinflammation, and α-synuclein expression, aggregation, and cell-to-cell transmission, as revealed in the present study. Consistent with this, most vulnerable dopaminergic neurons in the ventral tier of the SN show higher levels of AT1 receptor expression and lower levels of calbindin, which buffers raising in intracellular calcium^13,101,102^, as compared with more resistant neurons in the dorsal tier of the SN and other brain regions. Accordingly, neuroprotective effects of AT1 receptor blockers have been observed in several experimental and clinical studies^10,14,15,20^. Altogether reveals brain RAS dysregulation as a major mechanism involved in the progression of PD, and AT1 receptor inhibition and RAS modulation as a therapeutic target for PD.

## Methods

### Experimental design

Several sets of in vitro and in vivo experiments were designed to study the effects of the AngII/AT1 activity on α-synuclein aggregation and transmission. For the in vitro experiments (summarized in Fig. 1), we used different cell lines corresponding to dopaminergic neurons and major glial cells involved in dopaminergic degeneration: N27 dopaminergic neuronal cells, C6 astroglial cells and microglial cells (human HMC3 and mouse BV2). In addition, human kidney HEK293 cells were used as control cells. HEK293 can be used as a positive control for transfection because they have a very high transfection efficiency^32^, and they can also be used as a negative control for the effects of AngII, as HEK293 cells do not express AT1 and AT2 receptors^33^. In the vitro experiments, we first studied the efficiency of each cell type for α-synuclein aggregation and α-synuclein transmission. Then we studied the effects of AngII/AT1 activity using four in vitro experimental groups: untreated control cells, cells treated with AngII, cells treated with the AT1 blocker losartan, and cells treated with AngII and losartan. Losartan is usually chosen as an effective AT1 blocker for in vitro experiments in our laboratory and others. These experimental groups were used in two main types of experiments. First, to assess changes in the presence and quantity of aggregates, we used an α-synuclein aggregation model termed α-synuclein-T/V5-synphilin-1 (SynT/Sph1); this model (detailed below) has already been used for the assessment of the effects of different drugs on α-synuclein aggregation^36,38,40^. Second, the aggregate-like nature of the inclusions was determined with thioflavin S-staining (ThS). Third, to measure changes in the size of the aggregates we used both fluorescent microscopy and SDD-tricine western blotting (WB), and a modified version of bimolecular fluorescence complementation (BiFC) model that uses α-synuclein and synphilin-1 as the object proteins, by tagging α-synuclein with the VC terminus of venus protein and synphilin-1 with the VN terminus of venus protein;^34^ this model will be detailed below. Possible changes in levels of α-synuclein expression or phosphorylation were determined by WB. In addition, we studied possible α-synuclein transmission between different cell types and the effects of AngII on transmission by using co-cultures (via transwell), and by counting fluorescent cells using the BiFC method and flow cytometry (Fig. 1).

For the in vivo study of the effects of AngII/AT1 on α-synuclein aggregation, four treatment groups were established: mice treated with vehicle solution, mice treated with MPTP, mice treated with MPTP plus the AT1 blocker telmisartan and mice knockout for AT1 receptors treated with MPTP, as detailed below. Brains were extracted and then processed by immunohistochemistry for α-synuclein expression and by thioflavin S-staining (ThS) for aggregation. Image analysis was performed in the brain preparations as detailed below.

### In vitro experiments

To study the effects of AngII/AT1 on the formation of α-synuclein aggregates in different cells, we performed the SynT/Sph1 aggregation model^34–36^. This model can be used to study the effects of different treatments on α-synuclein aggregation in vitro in different cell types, as cells express a certain number of aggregate-like structures termed inclusions. For this procedure, we transfected the different cell lines concomitantly with plasmids encoding for α-synuclein fused to a truncated form of GFP termed SynT and plasmids encoding for V5-synphilin-1. Synphilin-1 is a protein that appears in the inner part of Lewy bodies and its interaction with α-synuclein leads to the formation of inclusions. V5 is a peptidic tag added to synphilin-1 to allow for easier detection by immunofluorescence^103^. Immunofluorescence was performed for the detection and quantification of the number of inclusions inside each cell (Fig. 1a). We observed that the efficiency of BV2 cells for the double transfection of the SynT/Sph1 model is too low, and we did not observe enough transfected cells to properly quantify different treatments. Therefore, we used HMC3 microglial cells for aggregation experiments, as their efficiency for double SynT/Sph1 transfection is much higher than that of BV2 cells. However, BV2 cells showed an adequate transfection rate for VN-α-Synuclein or α-Synuclein-VC and could be used for diffusion experiments (see below).

Different cell treatments may affect the formation of inclusions like how they would affect the formation of pathophysiological α-synuclein aggregates or the formation of Lewy bodies^39,104^. Therefore, treatments that increase the number of inclusions point to an increase in α-synuclein aggregation, and treatments that decrease the number of inclusions indicate a decrease in α-synuclein aggregation, which may have protective effects against the progression of PD. The aggregate-like nature of the inclusions was determined with Thioflavin (Th) (Fig. 1a). Thioflavin T and ThS are fluorescent dyes that bind to fibrillar amyloid structures, normally binding to beta-sheet structures in the proteins^105^. ThS has been extensively used for the study of amyloid-β, tau, and α-synuclein aggregates^106,107^.

The average inclusion size was measured using a combination of the SynT/Sph1 model with the BiFC method, as firstly developed by Lázaro and Outeiro^34,37^, by tagging α-synuclein with the VC terminus of venus protein and synphilin-1 with the VN terminus of venus protein (Fig. 1b). This leads to reconstitution of the fluorescent protein Venus when two proteins, each one carrying one half of venus, are close to each other and start to interact (Fig. 1b). This leads to the formation of fluorescent inclusions, whose size we measured directly by image analysis^80,108^. Treatments that decrease α-synuclein inclusion size may block the processes that lead to α-synuclein aggregation or break already formed aggregates into smaller ones. A decrease in both the number of inclusions and their size may point to a treatment that is protective against PD progression.

To determine α-synuclein diffusion between different cell types we performed a modified version of the BiFC method. We used BiFC to assess the selective transfer of α-synuclein between different cell types by having cells expressing α-synuclein fused with the VN fragment in a cell plate that is put into contact with cells fused with the VC fragment seeded in a transwell, or vice versa (Fig. 1c, d). Release and uptake of α-synuclein are necessary for the emission of fluorescence, as both halves of venus should be present inside the same cell and, at least, dimers of α-synuclein should be formed, enabling the interaction of both halves of venus, and the emission of fluorescence from that cell. Changes in the emission of fluorescence in one cell line, when co-cultured with a different cell line, point out changes in the release and uptake of α-synuclein. A decrease in the percentage of fluorescent cells points to a decrease in the uptake or an increase in the release of α-synuclein. Conversely, an increase in the percentage of fluorescent cells points to an increase in the uptake or a decrease in the release of α-synuclein. It has been proposed, even though it is debated, that the expression of VN-α-synuclein is higher than that of α-synuclein-VC, and that this system should not be used for the direct measurement of aggregation^109,110^. Therefore, we employed the system for the assessment of the transfer between cells and in the measurement of fluorescence areas, thus avoiding the direct measurement of aggregation. Additionally, taking this into account, we measured transfer changes in the VN and the VC fragments separately. As these techniques were not previously performed in C6, N27, BV2, and HMC3 cells, we additionally performed a series of controls to assess the validity of these techniques. Thus, for the SynT/Sph1 model, we performed immunofluorescence to check whether the inclusions observed are indeed α-synuclein aggregates, observing colocalization of both proteins. In addition, we used the aggregation marker Thioflavin S to further confirm the inclusions being indeed aggregates. Furthermore, for the SynT/Sph1 model and the BiFC method, we performed SDD-tricine western blots to confirm the formation of aggregates. This type of western blot breaks only partially the quaternary structure of proteins, thus allowing for the observation of oligomers and polymers that will appear at higher molecular weights than the monomeric protein. To check whether the effects observed in the BiFC experiments by the different treatments are due to changes in α-synuclein aggregation, and not in α-synuclein expression or phosphorylation, we performed western blots. To determine α-synuclein transmission and internalization, we performed co-cultures (via transwell) of the following cell lines: BV2 microglial cells, C6 astroglial cells, and N27 dopaminergic neuron cells. We performed all possible combinations between cell types and quantified fluorescent cells using the BiFC method and flow cytometry.

We also studied whether the effects of angiotensin II and the AT1 blocker losartan on α-synuclein aggregation may be mediated by two major mechanisms that are known to be triggered by AngII/AT1 activation, and that may affect α-synuclein aggregation (see Discussion): AngII/AT1 activation of the NADPH-oxidase complex with the subsequent increase in superoxide production, and the effects of AngII/AT1 activation on Ca^2+^ influx. We treated N27 dopaminergic neuron cells with either 2-Aminoethyl diphenylborinate (2-APB), which is known to inhibit Ca^2+^ influx through transient receptor potential 2 channels^111,112^, or with the NADPH-oxidase inhibitor apocynin^26,113^, before treatment with AngII. Two hours after transfection, cells were fed with fresh medium, and either 50 µM of 2-APB (Sigma, D9754) or 100 µM of apocynin (Thermo Scientific, 11458653) was added dropwise; half an hour later, the corresponding cells were treated with 100 nM AngII (Sigma Aldrich, A9525). Twenty-four and 48 h later, cells were treated again with the same drugs (i.e., apocynin or 2-APB, and AngII).

### In vivo experiments

Adult male C57BL6/J mice (8 weeks old at the beginning of the experiments) were used in the present study. All experiments were carried out in accordance with Directive 2010/63/EU and Directive 86/609/CEE and were approved by the animal experiment committee of the University of Santiago de Compostela (protocol 15005/15/002). All mice were anaesthetized by ketamine/xylazine prior sacrifice. Mice were wild-type (WT) or homozygous mice deficient for the type 1 angiotensin receptor (AT1 KO). Mice were divided into four groups comprising 6–14 animals per group. A group of WT mice (*n* = 14) received MPTP (Sigma, M0896) intraperitoneally for 5 days at a daily dose of 30 mg/kg. A second group of WT mice (*n* = 6) were orally treated with the AT1 blocker telmisartan (Sandoz, 5 mg/kg/day) starting 14 days before the beginning of the MPTP treatment and finishing 7 days after the end of the MPTP treatment. A group of AT1 KO mice were treated intraperitoneally for 5 days at a daily dose of 30 mg/kg MPTP. Telmisartan is one of the most effective AT1 blockers at crossing the blood–brain barrier, and low doses not affecting blood pressure are able to induce effects on brain RAS in vivo^10,43^. Finally, a control group of WT mice were treated with saline for 5 days (*n* = 13). Brains were extracted and then processed for WB, α-synuclein immunohistochemistry, and thioflavin S-staining for aggregation. Brain sections were studied using Image analysis as described below.

### Cell cultures

Human embryonic kidney (HEK293) and human microglial clone (HMC3) cells were cultured in Dulbecco’s modified Eagle medium (DMEM; 10-013-CV, Falcon Corning) with 10% inactivated fetal bovine serum (FBSi), 1% penicillin/streptomycin and 1% fungizone at 37 °C and 5% CO_2_ in a humidified incubator. C6 rat astroglial cells were cultured in a 1:1 mixture of DMEM and Ham’s F12 medium (10-092-CV, Falcon Corning) with 10% FBS, 1% penicillin/streptomycin, and 1% fungizone at 37 °C and 5% CO_2_ in a humidified incubator. BV2 mouse microglial cells and N27 rat dopaminergic neuron cells were cultured in Roswell Park Memorial Institute (RPMI) medium (R8758-1640) both supplemented with 10% FBS, 1% penicillin/streptomycin and 1% fungizone at 37 °C and 5% CO_2_ in a humidified incubator. Twenty-four hours before transfection, ~100,000 cells were plated per well in a 12-well plate (Falcon, ref 353043). Transfection was performed on the plate. For HEK293 and C6 cell lines transfection was performed with TRANSIT-X2 transfection reagent (Mirus Bio, Madison, USA) according to the following protocol: 1 µg of total DNA and 2 µl of TRANSIT-X2 were added to 100 µl of DMEM allowing this mix 20 min of incubation time. Otherwise, the plate containing HMC3 was transfected with magnetic nanoparticles by using Glial Mag transfection reagent (OZBioscience, GL00500) according to the following protocol: 1 µg of total DNA and 3.5 µl of Glial Mag were added to 100 µl of DMEM allowing this time 30 minutes of incubation time. Then, Glial Boost reagent was added to this mix (OZBioscience, GL00500) according to the following protocol: 3 µl of Glial Boost for each µg of DNA added. The resulting mixture was added dropwise to the cells, and the plate was gently rocked. Mixtures containing the Glial Mag reagent and HMC3 cells were subjected to magnetofection using a Super Magnetic Plate (OZBioscience, MF10000) for 45 min to improve the introduction of the DNA into the cells. Cells were kept in an incubator at 37 °C and 5% CO_2_ for an additional 48 h.

### Protein diffusion and cell treatments

For the study of α-synuclein diffusion, transwell permeable transparent PET membrane supports for six-well plate (Falcon 353090; pore diameter of 400 nm and a nominal width of 10 μm) were used. The transwells were placed before seeding the cells. Cells were seeded on them, and once cells were seeded and grown, they were transfected either with the BiFC VN-α-synuclein construct or with the α-synuclein-VC construct. Approximately 72 h after transfection, transwells, and plate wells media were changed, and the transwells were placed in the corresponding plate well so that α-synuclein diffusion occurred. This diffusion process was left for 72 h, and then the cells were collected for the corresponding experiment.

Two hours after transfection, cells were fed with fresh medium, and 10 µM of losartan potassium (Sigma Aldrich, 61188-100MG) was added dropwise, half an hour later, the corresponding cells were treated with 100 nM AngII (Sigma Aldrich, A9525). At 24 h and 48 h later, cells were treated again. For α-synuclein diffusion analyses, treatments were carried out right after transferring the transwell to its corresponding well to perform the α-synuclein diffusion process. 24 h after the third treatment, the cells were processed for their corresponding experiment.

### Immunofluorescence of cell cultures for inclusion quantification

Cells transfected with VN-α-synuclein and α-synuclein-VC were washed with Dulbecco’s phosphate-buffered saline (DPBS; pH 7.4) and fixed with 4% paraformaldehyde (PFA) for 20 min. After fixation, cells were washed three times, incubated for 10 min with the DNA-binding dye Hoechst 33342 (1:8000 in DPBS), washed three times with DPBS, and kept until analysis. Cells transfected with α-Synuclein-T and V5-synphilin-1 were washed with Dulbecco’s phosphate-buffered saline (DPBS; pH 7.4) and fixed with 4% paraformaldehyde for 20 min. After fixation with 4% PFA, cells were blocked and permeabilized for 1 h at room temperature with 1% BSA, 2% normal donkey serum (NDS, Sigma), and 1.5% Triton-X-100 in DPBS. Following permeabilization, cells were incubated overnight at 16 °C with an anti-α-synuclein antibody MJFR1 (1:1500; Abcam, Rabbit, ab138501) in DPBS with 1% BSA and 2% NDS. The cultures were rinsed with DPBS and then incubated for 2 h with the fluorescent secondary antibody Alexa Fluor 488-conjugated donkey anti-rabbit IgG (1:200; Thermo Fisher Scientific, A-21206). Then, cultures were also incubated for 10 min with the DNA-binding dye Hoechst 33342 (1:8000 in DPBS). Finally, cells were washed three times with DPBS and kept until analysis.

### Immunofluorescence of cell cultures for inclusion characterization

Cells transfected with α-Synuclein-T and V5-synphilin-1 were grown on glass coverslips. These cells were washed with Dulbecco’s phosphate-buffered saline (DPBS; pH 7.4), incubated with 0.01% Thioflavin S (ThS) (Sigma Aldrich, T1892) for 8 min, and fixed with 4% paraformaldehyde for 20 min. After that, blocking and permeabilization of cells were performed for 1 h at room temperature with 1% BSA, 2% normal donkey serum (NDS, Sigma), and 1.5% Triton-X-100 in DPBS. Following permeabilization, cells were incubated overnight at 16 °C with anti-α-synuclein antibody MJFR1 (1:1500) and anti-V5 antibody (1:3000; Abcam, Goat, ab9137) in DPBS with 1% BSA and 2% NDS. The cultures were rinsed with DPBS and then incubated for 2 h with the following fluorescent secondary antibodies: Alexa Fluor 568-conjugated donkey anti-rabbit IgG (1:200; Thermo Fisher Scientific, A10042) and Alexa Fluor 633-conjugated donkey anti-goat IgG (1:200; Thermo Fisher Scientific, A-21082). Then, cultures were also incubated for 10 min with the DNA-binding dye Hoechst 33342 (1:8000 in DPBS; Sigma Aldrich, B2261). Cells were washed three times with DPBS and kept until analysis. Finally, the coverslips were mounted with Immu-Mount (Thermo Shandon), and fluorescence was analyzed by thunder microscopy (Leica DM4B) and a Leica TCS SP5 confocal microscope.

### Immunofluorescence of mouse brains

Animals were anesthetized by ketamine/xylazine prior to sacrifice and then perfused, firstly with 0.9% saline, and then with cold 4% paraformaldehyde in 0.1 M phosphate buffer, pH 7.4. Brains were removed, washed, and cryoprotected in the same buffer containing 20% sucrose, and finally cut on a freezing microtome with a thickness of 30 μm. First, sections were incubated for 1 h at room temperature with a 0.1% solution of the aggregation marker ThS. Then, sections were washed three times with 20 mM KPBS and incubated for 1 h at room temperature with 20 mM KPBS containing 5% BSA, 5% normal serum, and 0.3% Triton X10. Then, sections were incubated overnight at 4 °C with the primary antibodies. Sections were processed with rabbit α-synuclein MJFR1 (1:500) or mouse α-synuclein LB509 (1:500; Abcam, ab27766) primary antibodies (depending on the second primary antibody used for colocalization studies), as well as mouse polyclonal antibodies to Glial Fibrillary Acidic Protein (GFAP; 1:1000; EMD Millipore, MAB360) as a marker of astrocytes, ionized calcium-binding adapter molecule 1 (IBA-1 EPR6136; 1:100; Abcam, ab178680) as a marker of microglia, and mouse monoclonal antibodies against Tyrosine Hydroxylase (TH; 1:1000; Sigma Aldrich, T2928) as a marker of dopaminergic neurons. The next day, the sections were washed three times with 20 mM KPBS, and subsequently incubated, firstly for 2 h with the corresponding secondary antibodies: Alexa fluor 568-conjugated donkey anti-rabbit (1:100; Invitrogen by Thermo Fisher, A10042), Alexa fluor 647 conjugated donkey anti-mouse (1:100; Invitrogen by Thermo Fisher, A-31571), Alexa fluor 568-conjugated donkey anti-mouse (1:100; Invitrogen by Thermo Fisher, A10037) and Alexa fluor 647 conjugated donkey anti-rabbit (1:100; Invitrogen by Thermo Fisher, A-31573), and then for 10 min at room temperature with Hoechst 33342 (3 × 10−5 M in KPBS). Sections were mounted on gelatin-coated slides and covered with coverslips using Immu-Mount (Thermo Shandon, 10622689) as a mounting medium, and analyzed by thunder microscopy (Leica DM4B) and a Leica TCS SP5 confocal microscope.

### Microscopy quantification

For inclusion quantification and size analysis, pictures were taken on a Leica DMI6000B fluorescence microscope at ×40 with an HC PL Fluotar L ×40/0.60 dry objective (reference 11506201) and a DFC360FX-31202708 camera with 460, 527, and 630 nm filters simultaneously. At least 30 pictures were taken for each treatment and experiment. Pictures were subsequently analyzed in ImageJ software (https://imagej.nih.gov/ij/). The labeling of cell line cultures and mouse brain immunofluorescence was visualized, and pictures were taken on a Leica DM4B microscope with an HC PL fluotar ×40 objective and a Leica-K5-14401204 camera with 460, 527, 630, and 700 nm filters simultaneously.

For determination of the size of α-synuclein inclusions in cell cultures, pictures were analyzed with ImageJ’s software. For each image, a color threshold of the image was performed and adjusted. The resulting image was used to analyze the particle size directly with the analyze particles plug-in. First, the positive threshold for the particles was established, and then we assigned a lower limit of 0.016 μm^2^ in the analyze particles plug-in from ImageJ. ImageJ software itself assigns a value for the areas measured based on data generated by the microscope through Leica Application Suite X (LAS X, Leica). For quantification of α-synuclein inclusions of cell cultures, cells transfected with α-synuclein were scored based on the α-synuclein inclusion pattern and further classified based on the presence or absence of inclusions and the data were expressed as percentage of cells with inclusions.

For quantification of α-synuclein inclusions of mouse brains, the area quantification for each antibody-ir and for the binding to ThS was estimated by measurement of particle area via LAS X and ImageJ, as follows: at least 30 sections were pictured, and the images we subjected to a de-blurring process via Thunder Imaging in LAS X. The image threshold was adjusted in ImageJ and the resulting image was used to analyze the particle size directly with the analyze particles plug-in. The area value obtained was expressed as a percentage of the total picture area.

### Western blot analysis

Cellular protein levels of α-synuclein, and β-actin were analyzed by WB. The protein was extracted by cell homogenization with RIPA buffer with protease inhibitor cocktail, phosphatase inhibitor cocktail, and PMS. After protein extraction, samples were sonicated for 3 seconds at 10% amplitude and 25 °C temperature. After that, the total protein concentration was calculated by the Pierce BCA protein assay. Equal amounts of protein were separated by 12% bis-tris polyacrylamide gel and transferred to the nitrocellulose membrane. The membranes were incubated overnight with primary antibodies against α-synuclein (Ab80627, Abcam; mouse; 1:2000) and phosphorylated α-synuclein S129 (Ab59264, Abcam; rabbit 1:2000). The fluorescent secondary antibodies were donkey anti-mouse (IRDye 680LT, LI-COR; 926-68022; 1:20000) and donkey anti-rabbit (IRDye 800CW, LI-COR; 926-32213; 1:20000). Immunofluorescence was visualized with a fluorescence detection system (Molecular Imager ChemiDoc MP imaging System, BioRad). Blots were incubated with the antibody β-actin (A2228, Sigma; 1:10,000) as loading control. Protein expression was measured by densitometric analysis of each corresponding band using ImageJ’s software and expressed relative to the β-actin band value. The data were normalized with the control groups (100%) corresponding to each study group to compare the possible variabilities between both groups. Semi-denaturing tricine (SDD-tricine) WB was performed to assess the presence and relative size of α-synuclein aggregates. Semi-denaturing WB, specifically a variation known as SDD-AGE WB has been widely used for the estimation of amyloid beta aggregate size. In these types of WB, proteins are not completely denaturalized, thus partially keeping their tertiary and quaternary structure^41,114,115^. These types of WB suppose a middle ground between standard SDS-PAGE WB and native WB, allowing us to check the presence, relative size, and packing strength of protein aggregates, which can appear either as smears or thick bands of different molecular weights. The protocol followed is a modification of a previously proposed one^41^. In brief, cell or mouse brain tissue homogenates were loaded into 10% tricine gels. The electrophoresis was performed with a running buffer containing 0.1 M Tris base, 0.1 M tricine, and 0.1% SDS. Gels were then transferred to nitrocellulose membranes. The membranes were incubated overnight with 1:2000 mouse anti-aggregated-aSyn (Ab80627, Abcam; mouse) at 4 °C. Following three washes with TBS-Tween, membranes were incubated for two additional hours with the corresponding HRP-conjugated secondary antibody (GE Healthcare). After incubation with a secondary antibody, membranes were washed three times with TBS-Tween and developed in a chemiluminescence system (Molecular Imager ChemiDoc XRS System, BioRad).

Mouse nigral tissue was homogenized in 10 mM Tris-HCl (pH = 7.4) containing 0.1% SDS, protease inhibitor cocktail, and phosphatase inhibitor cocktail and centrifuged at 14,000 rpm for 20 min at 4 °C. After homogenization, samples were incubated at 37 °C for 1 h 30 min and then centrifuged. Total protein concentration was measured by the Pierce BCA protein assay kit. Samples were aliquoted into three different groups: one for pK digestion, a second one for pSer129 blotting, and a third one for SDD-tricine semi-denaturing western blot.

For pK digestion, mouse nigral tissue homogenates containing 20 mg of total protein were digested with 3 µg/ml pK (proteinase K from Trichirachum album P6556, Sigma) at 37 °C for some seconds, 5, 15, and 30 minutes. After each period, pK was neutralized with a loading buffer. Following this, samples were boiled at 95 °C for 5 minutes loaded into a 12% bis-tris polyacrylamide gel, and transferred to a nitrocellulose membrane. Membranes were incubated overnight with an anti-α-synuclein antibody (Ab80627, Abcam; mouse; 1:2000) and anti-β-actin antibody (A2228, Sigma; 1:10000) as a loading control. For pSer129 blotting, mouse nigral tissue homogenates were boiled and loaded into 12% bis-tris polyacrylamide gels. Membranes were incubated overnight with an anti-pSer129-α-synuclein antibody (Ab59264, Abcam; rabbit 1:2000) and anti-β-actin antibody (A2228, Sigma; 1:10000) as loading control. For SDD-tricine blotting, samples containing 20 mg of total protein were directly loaded into 10% tricine gels, as described above. For each experiment, all blots or gels derived from the same experiment and were processed in parallel. Uncropped membranes are available as supplementary information.

### Triton-X and SDS solubility

To determine Triton-X and SDS-soluble fractions from total protein, cells were collected in a buffer containing 1% of Triton-X-100 in 50 mM Tris and 150 mM NaCl (pH 7.6) with protease and phosphatase inhibitors, and the resulting solutions were left at room temperature for at least 15 minutes. After this, the solutions were sonicated three times for 3 seconds at 10% intensity. After sonication, samples were centrifuged at 14,000 × *g* for 10 minutes, and pellets and supernatants were collected. Pellets were dissolved again in SDS buffer containing 1% SDS in 50 mM Tris and 150 mM NaCl (pH 7.6) with protease and phosphatase inhibitors. Samples were centrifuged again at 14,000 × *g* for 10 minutes, and the new resulting pellets and supernatants were collected. Pellets were finally dissolved in 100 µl of SDS buffer. All fractions obtained were boiled at 95° C for 5 minutes and loaded into 12% polyacrylamide gels for SDS-PAGE western blot.

For native (non-denaturing membranes), samples were directly stained and loaded into Native PAGE pre-cast gels (Invitrogen), and electrophoretic separation was performed according to the manufacturer’s instructions.

### SiRNA transfection and treatments

For SiRNA transfection, N27 cells already transfected either for the BiFC or for SynT model were left to grow for 24 hours or until reaching 80% confluence. After this, the cells were transfected with the gp91phox/CYBB/NOX2 siRNA (Santa Cruz, sc-61838) following the manufacturer’s instructions. Twenty-four hours later, cells were treated with 100 nM AngII. Twenty-four hours later, cells were processed for the corresponding experiment. The silencing of the gene was confirmed by western blotting following the manufacturer’s recommendations.

### NADPH-oxidase activity measurements

N27 cells were collected in krebs-hepes buffer and homogenized. The resultant suspension was assayed for protein concentration using the Pierce BCA Protein Assay Kit (Thermo Fisher Scientific). NADPH-oxidase activity was measured in 30 μg of extracted proteins in the presence of lucigenin (5 × 10^−6^ mol/L; Sigma) and NADPH (10^− 4^ mol/L; Sigma). Measurements were performed with an Infinite M200 multiwell plate reader (Tecan, Switzerland). No enzymatic activity was detected in the absence of NADPH.

### Calcium measurements

Changes in intracellular Ca^2+^ were determined using the Fluo-4 AM Ca^2+^ sensor (Thermo Fisher Scientific). Control and treated N27 cells were incubated in 5 µM Fluo-4 AM at 37 °C for 60 min. After washing the cells with calcium-free buffer, fluorescence was analyzed using a widefield microscope (Leica DMI6000B) (488 nm excitation, 520 nm emission). To study intracellular Ca^2+^ changes, each cell was defined as a region of interest (ROI) using ImageJ. Changes in fluorescence intensity in each ROI corresponded to intracellular Ca^2+^ changes and were measured as normalized relative fluorescence (∆F) relative to basal fluorescence (recording period before AngII addition).

### Flow cytometry

The cells were first trypsinized to lift them, and then they were neutralized with FBSi medium. The cell suspension was centrifuged, the supernatant discarded, and the pellet was resuspended in phosphate-buffered saline (PBS) + 5% FBSi. 10,000 events were counted for each experiment in a BD Accuri C6 Plus Personal Flow Cytometer. Normalization was performed by assigning a value of 100 to the average of the fraction of positive (i.e., fluorescent) cells in the mixes between cells of the same type. Subsequently, mixes between different cell types and AngII treatments were assigned their corresponding percentile value in relation to the mixes between cells of the same type.

The cell population was chosen from a preliminary FSC/SCC gating (supplementary Fig. 2). Gated cells were further divided into fluorescence-positive and negative cells. Positive cells were defined as cells with a venus fluorescence intensity higher than 98,000, 100,000, or 120,000 RFU, based on the fluorescence intensity values of the negative and positive controls for each cell type in all experiments (supplementary Figs. 3–4). For transwell experiments, cells seeded inside transwells and cells seeded on plate wells were analyzed separately. Positive and negative cells were analyzed as mentioned (supplementary Figs. 5–7).

### Statistical analysis

The normality of populations and homogeneity of variances were tested before each analysis of variance using the Kolmogorov–Smirnov test. For multiple comparisons, when the dataset passed the normality test, a one-way analysis of variance (ANOVA) was performed. ANOVA tests were followed by the corresponding post hoc test based on the normality and homogeneity of the data. Pairwise comparisons were done by non-paired *t* tests or the corresponding non-parametric tests. Differences were considered statistically significant at *p* ≤ 0.05. All data were obtained from at least four independent experiments and were expressed as means ± standard error of the mean. Statistical analyses and scatter dot plot graphs were performed with GraphPad Prism 8.

### Reporting summary

Further information on research design is available in the Nature Research Reporting Summary linked to this article.

### Supplementary information

Supplementary information

Checklist

Supp movie 1 Control striatum

Supp movie 2 MPTP striatum

Supp movie 3 Control SN

Supp movie 4 MPTP SN

Supp movie 5 BiFC C6 transwell

## Data Availability

The data supporting the conclusions of this article are included within the article and its additional files. Additional data are available from the corresponding author upon request.

## Code availability

Statistical analysis was performed using GraphPad Prism Version 8.0.2, and GraphPad syntax used for the analysis is available from the corresponding author upon request.
