# Supplementary material for: Angiotensin type 1 receptor activation promotes neuronal and glial alpha-synuclein aggregation and transmission
Source: NPJ Parkinsons Dis. 2024 Feb 17;10:37. doi: 10.1038/s41531-024-00650-0 (PMC10874459; doi:10.1038/s41531-024-00650-0)

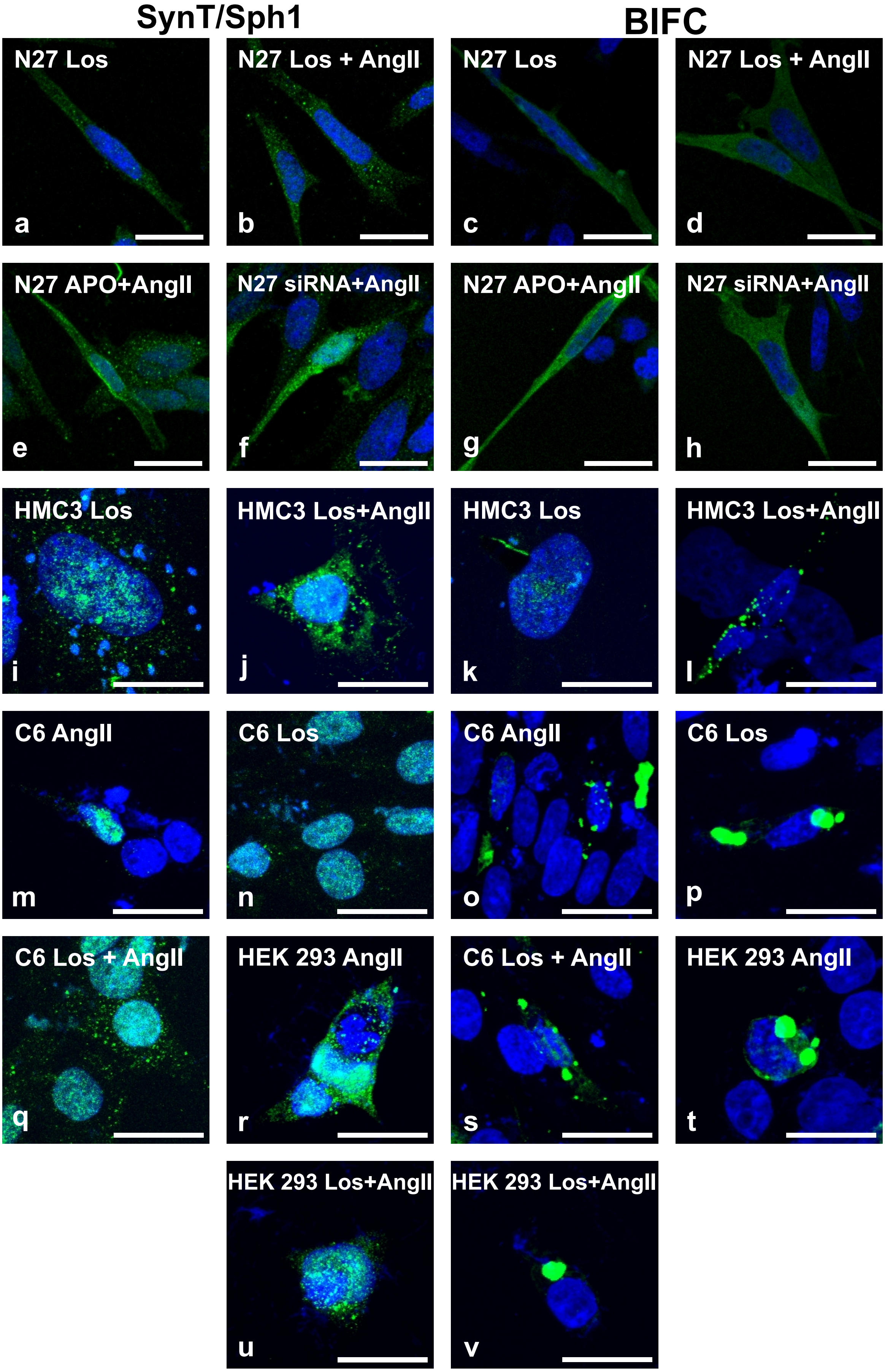

Supplementary Figure 1. Confocal pictures of cells analyzed for the number of inclusions and inclusion size representative of treatments that did not induce significant changes relative to controls shown in Fig. 3a-l and 5e-h. Los: Losartan. APO: Apocynin. Scale bar: 20 µm.

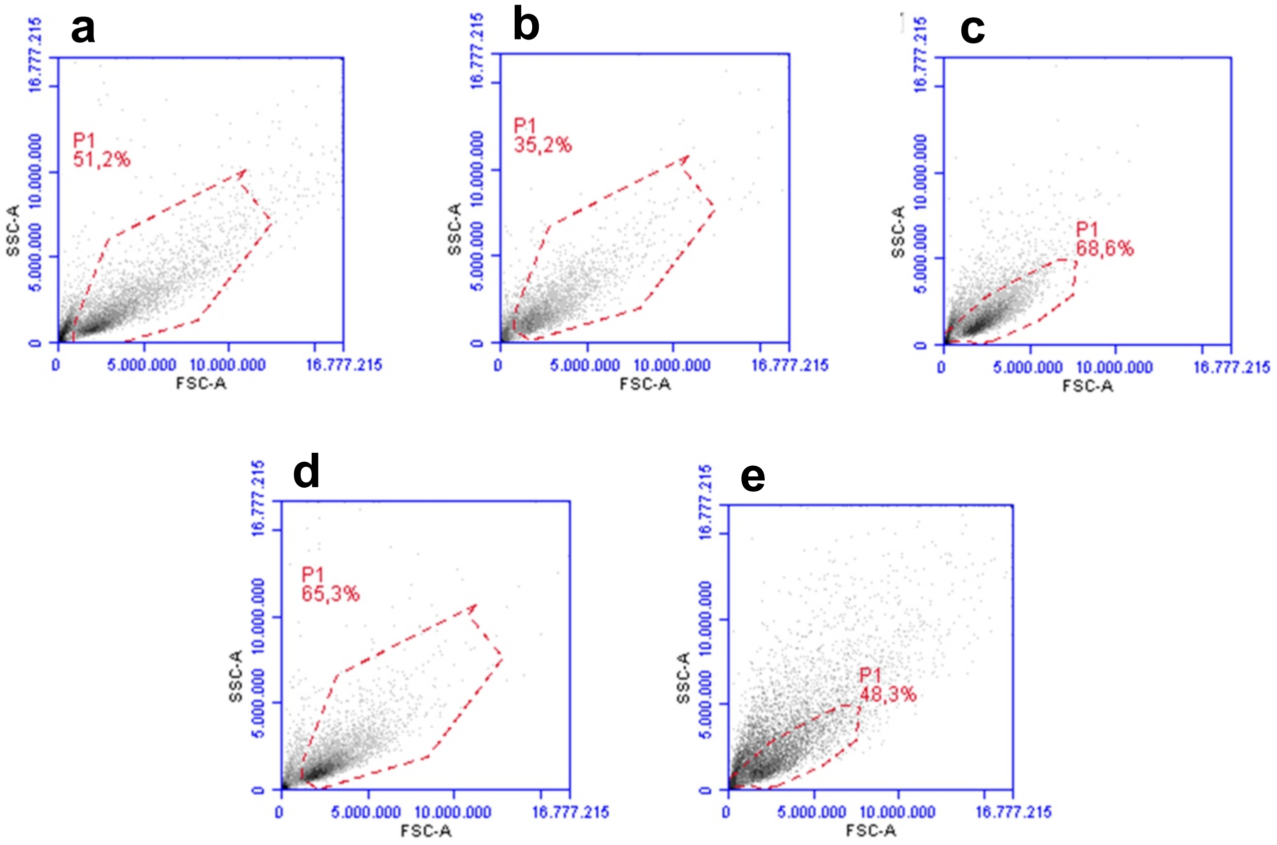

Supplementary Figure 2. Representative flow cytometry dot-plots showing the initial gate for the following cell lines: N27 (**a**), HMC3 (**b**), BV2 (**c**), C6 (**d**) and HEK293 (**e**). Histograms of the supplementary figures S2-S6 follow the gating strategies shown in this figure for each cell line.

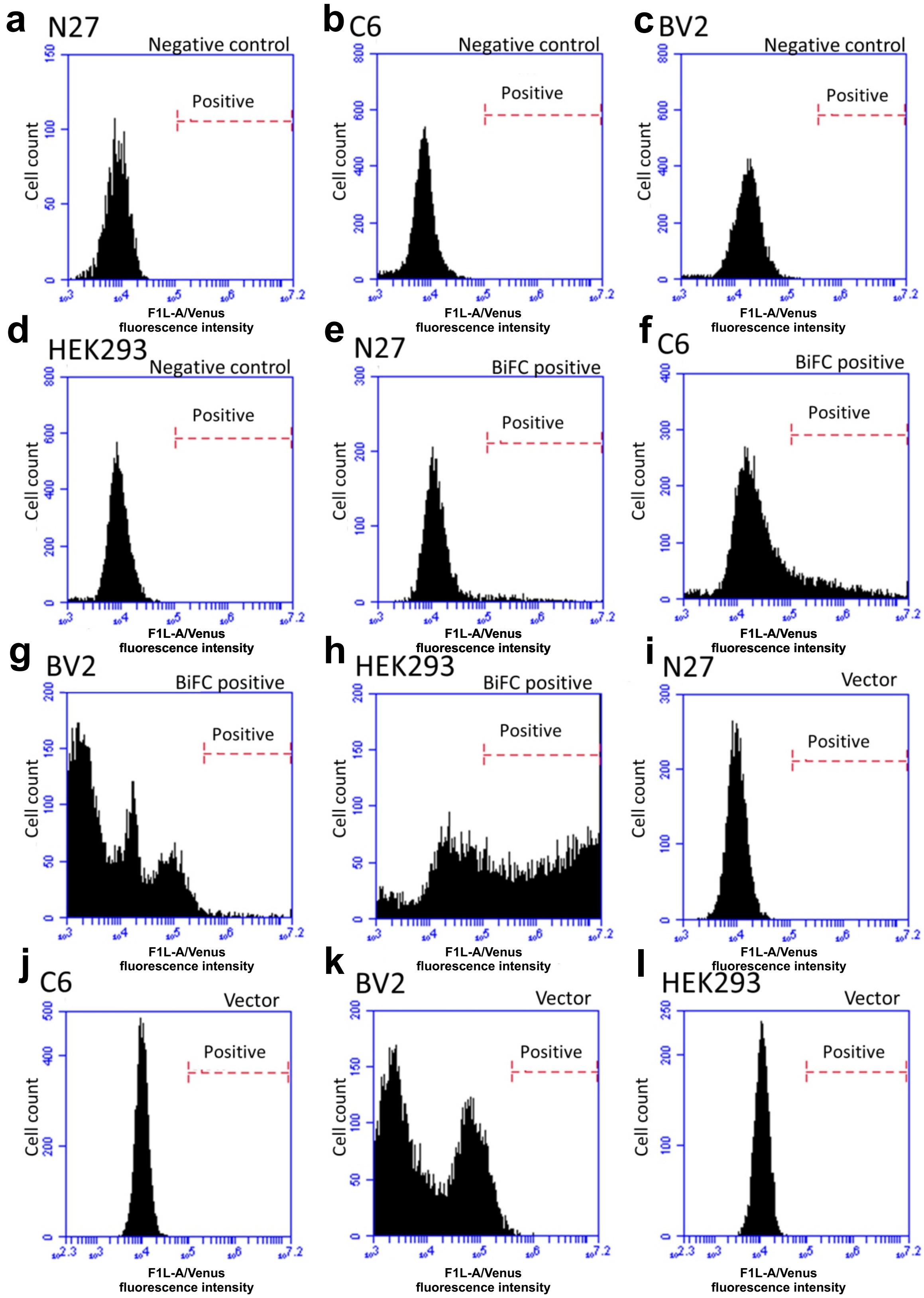

Supplementary Figure 3. (**a-d**) Representative flow cytometry histograms for negative N27, C6, BV2 and HEK293 cells.10000 events analyzed. (**e-h**) Representative flow cytometry histograms for N27, C6, BV2 and HEK293 cells transfected simultaneously with VN-α-Synuclein and α-Synuclein-VC. 10000 events analyzed. (**i-l**) Representative flow cytometry histograms for N27, C6, BV2 and HEK293 cells transfected with empty vector.10000 events analyzed.

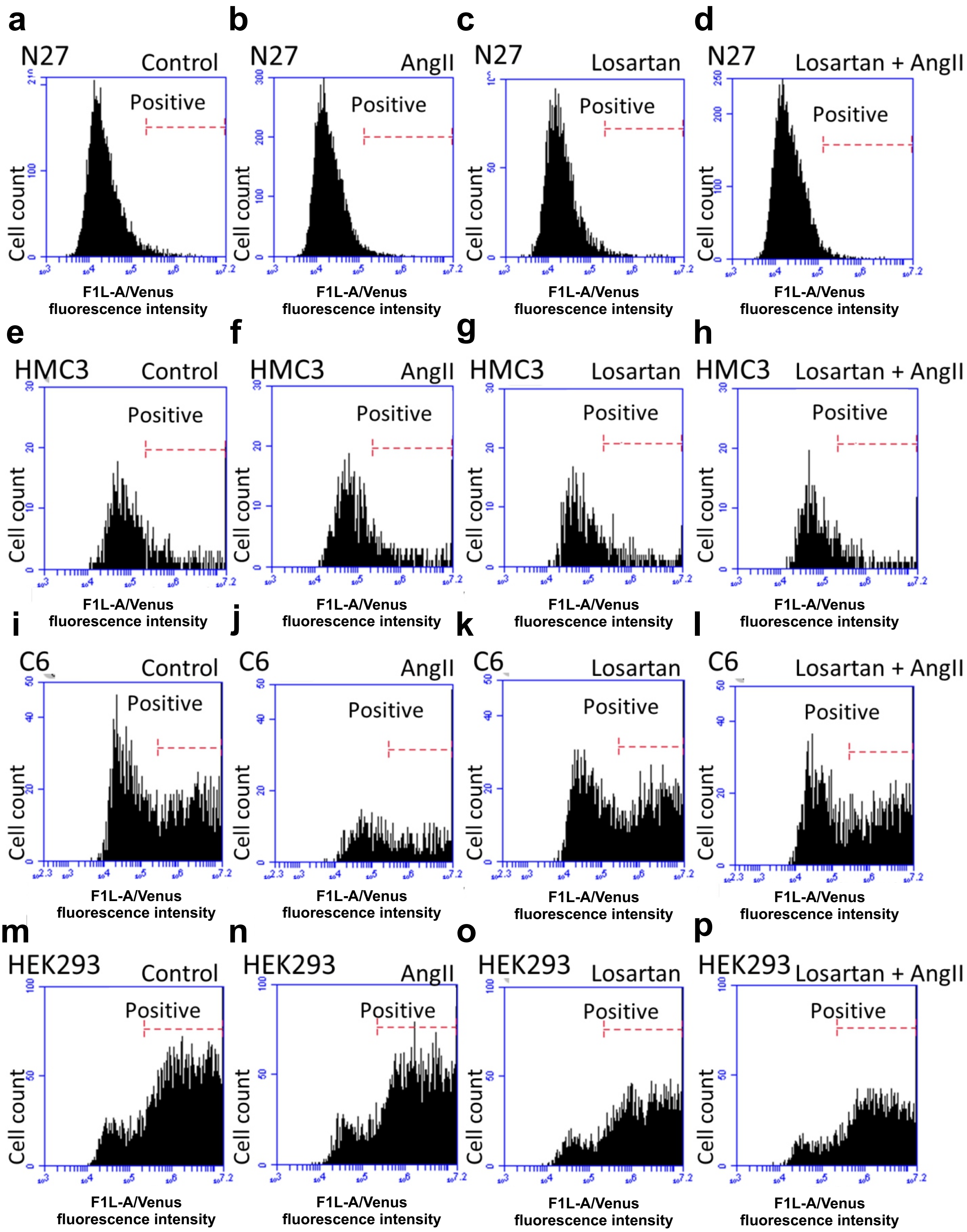

Supplementary Figure 4. Representative flow cytometry histograms for BiFC positive cells: untreated BiFC positive N27 cells (**a**); N27 cells treated with AngII (**b**); N27 cells treated with losartan (**c**); N27 cells treated with losartan and AngII (**d**); untreated BiFC positive HMC3 cells (**e**); HMC3 cells treated with AngII (**f**); HMC3 cells treated with losartan (**g**); HMC3 cells treated with losartan and AngII (**h**); untreated BiFC positive C6 cells (**i**); C6 cells treated with AngII (**j**); C6 cells treated with losartan (**k**); C6 cells treated with losartan and AngII (**l**); untreated BiFC positive HEK293 cells (**m**); HEK293 cells treated with AngII (N); HEK293 cells treated with losartan (**o**); HEK293 cells treated with losartan and AngII (**p**). On each analysis, 10000 events were analyzed.

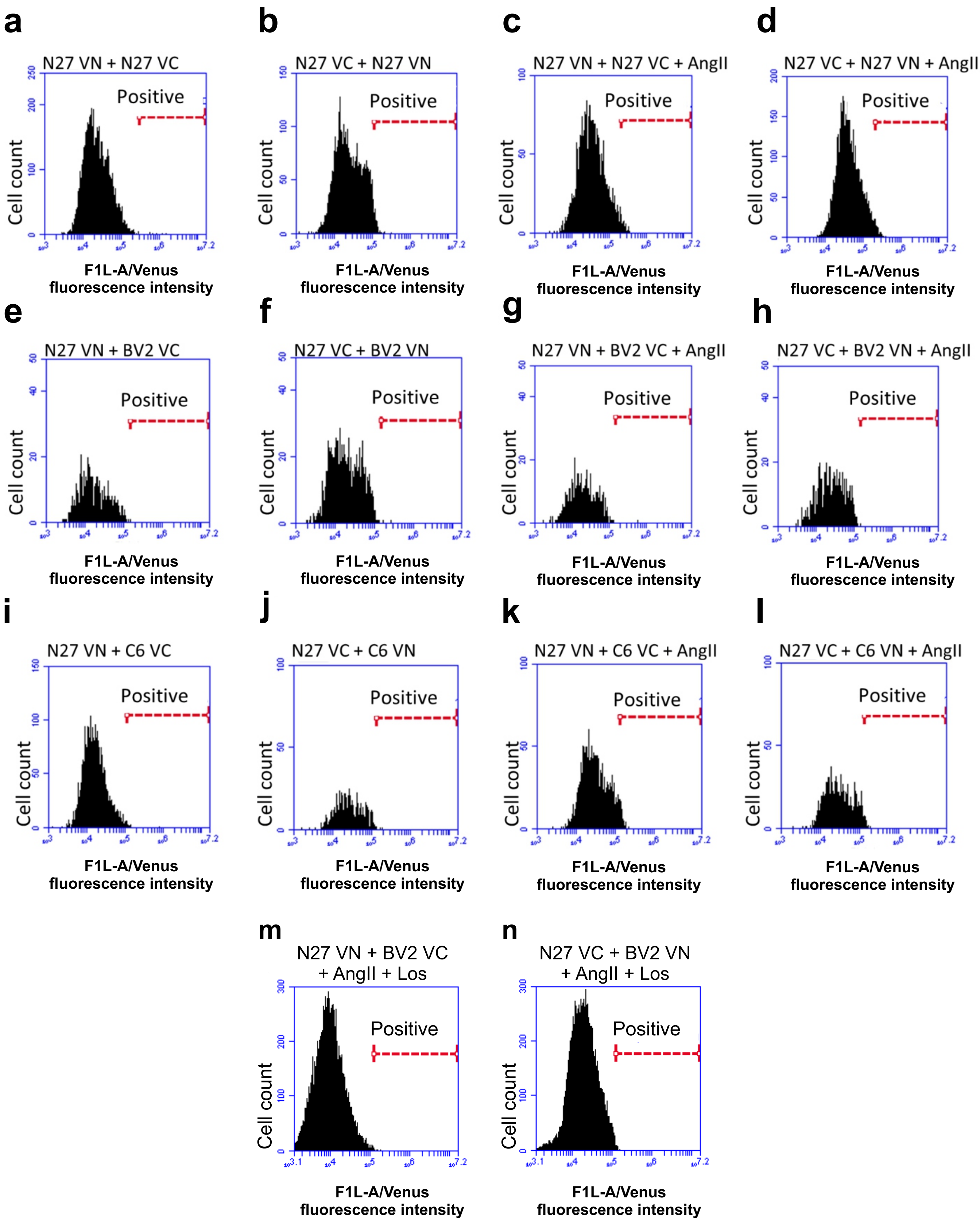

Supplementary Figure 5. Representative flow cytometry histograms for the following cells: (**a**) VN N27 cells for the co-culture between VN N27 cells and N27 VC cells. (**b**) N27 VC cells for the co-culture between VN N27 cells and N27 VC cells. (**c**) VN N27 cells for the co-culture between VN N27 cells and N27 VC cells after AngII treatment. (**d**) N27 VC cells for the co-culture between VN N27 cells and N27 VC cells after AngII treatment. (**e**) VN N27 cells for the co-culture between VN N27 cells and BV2 VC cells. (**f**) N27 VC cells for the co-culture between VN BV2 cells and N27 VC cells. (**g**) VN N27 cells for the co-culture between VN N27 cells and BV2 VC cells after AngII treatment. (**h**) N27 VC cells for the co-culture between VN BV2 cells and N27 VC cells after AngII treatment. (**i**) VN N27 cells for the co-culture between VN N27 cells and C6 VC cells (**j**) N27 VC cells for the co-culture between VN C6 cells and N27 VC cells. (**k**) VN N27 cells for the co-culture between VN N27 cells and C6 VC cells after AngII treatment. (**l**) N27 VC cells for the co-culture between VN C6 cells and N27 VC cells after AngII treatment. (**m**) VN N27 cells for the co-culture between VN N27 cells and BV2 VC cells after losartan and AngII treatment. (**n**) N27 VC cells for the co-culture between VN BV2 cells and N27 VC cells after losartan and AngII treatment.10000 events analyzed.

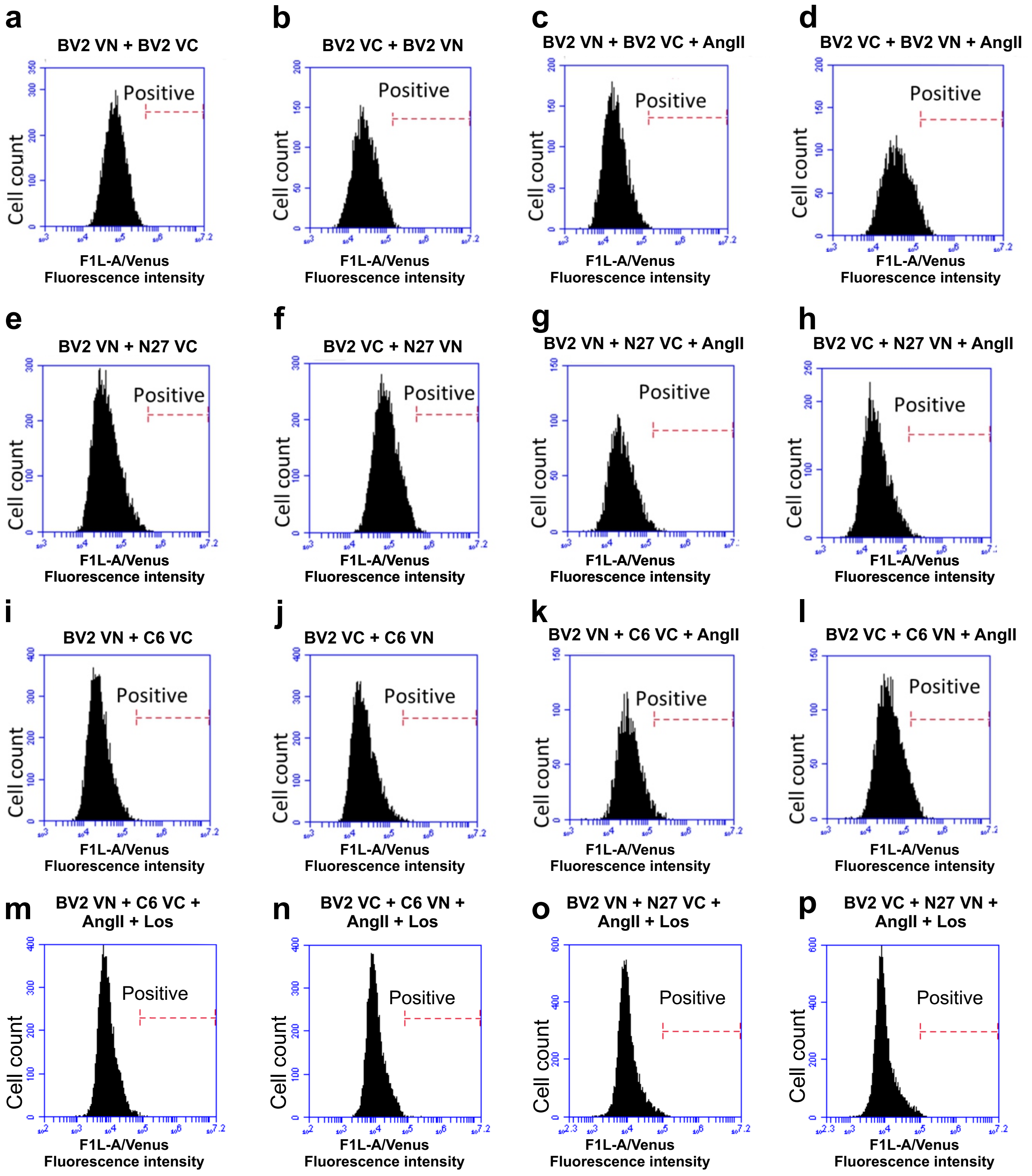

Supplementary Figure 6. Representative flow cytometry histograms for the following cells: (**a**) VN BV2 cells for the co-culture between VN BV2 cells and BV2 VC cells. (**b**) BV2 VC cells for the co-culture between VN BV2 cells and BV2 VC cells. (**c**) VN BV2 cells for the co-culture between VN BV2 cells and BV2 VC cells after AngII treatment. (**d**) BV2 VC cells for the co-culture between VN BV2 cells and BV2 VC cells after AngII treatment. (**e**) VN BV2 cells for the co-culture between VN BV2 cells and N27 VC cells. (**f**) BV2 VC cells for the co-culture between VN N27 cells and Bv2 VC cells. (**g**) VN BV2 cells for the co-culture between VN BV2 cells and N27 VC cells after AngII treatment. (**h**) BV2 VC cells for the co-culture between VN N27 cells and BV2 VC cells after AngII treatment. (**i**) VN BV2 cells for the co-culture between VN BV2 cells and C6 VC cells. (**j**) BV2 VC cells for the co-culture between VN C6 cells and BV2 VC cells. (**k**) VN BV2 cells for the co-culture between VN BV2 cells and C6 VC cells after AngII treatment. (**l**) BV2 VC cells for the co-culture between VN C6 cells and BV2 VC cells after AngII treatment. (**m**) VN BV2 cells for the co-culture between VN BV2 cells and C6 VC cells after losartan and AngII treatment. (**n**) BV2 VC cells for the co-culture between VN C6 cells and BV2 VC cells after losartan and AngII treatment. (**o**) VN BV2 cells for the co-culture between VN BV2 cells and N27 VC cells after losartan and AngII treatment. (**p**) BV2 VC cells for the co-culture between VN N27 cells and BV2 VC cells after losartan and AngII treatment. 10000 events analyzed.

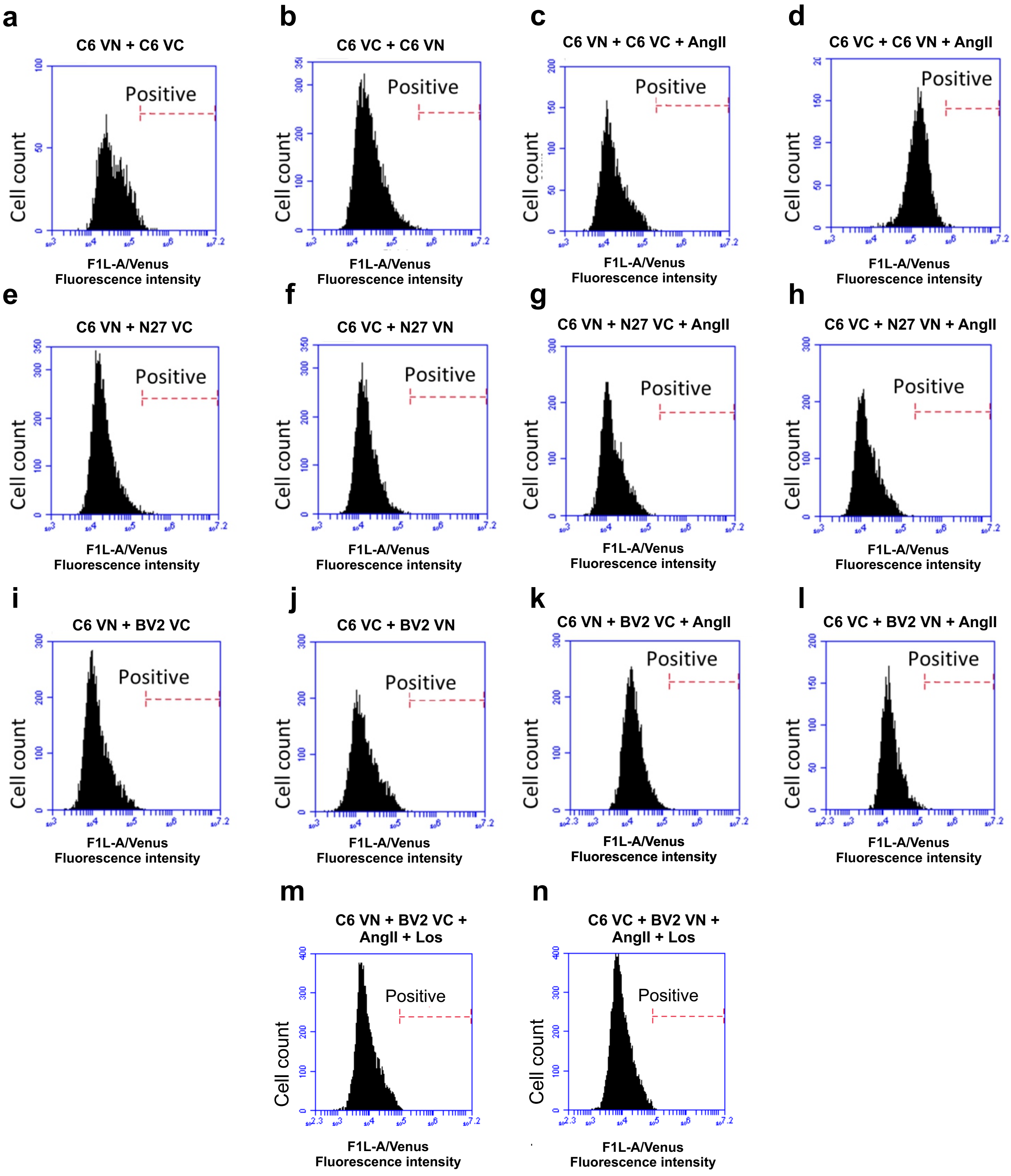

Supplementary Figure 7. Representative flow cytometry histograms for the following cells: (**a**) VN C6 cells for the co-culture between VN C6 cells and C6 VC cells. (**b**) C6 VC cells for the co-culture between VN C6 cells and C6 VC cells. (**c**) VN C6 cells for the co-culture between VN C6 cells and C6 VC cells after AngII treatment. (**d**) C6 VC cells for the co-culture between VN C6 cells and C6 VC cells after AngII treatment. (**e**) VN C6 cells for the co-culture between VN C6 cells and N27 VC cells. (**f**) C6 VC cells for the co-culture between VN N27 cells and C6 VC cells. (**g**) VN C6 cells for the co-culture between VN C6 cells and N27 VC cells after AngII treatment. (**h**) C6 VC cells for the co-culture between VN N27 cells and C6 VC cells after AngII treatment. (**i**) VN C6 cells for the co-culture between VN C6 cells and BV2 VC cells. (**j**) C6 VC cells for the co-culture between VN BV2 cells and C6 VC cells. (**k**) VN C6 cells for the co-culture between VN C6 cells and BV2 VC cells after AngII treatment. (**l**) C6 VC cells for the co-culture between VN BV2 cells and C6 VC cells after AngII treatment. (**m**) VN C6 cells for the co-culture between VN C6 cells and BV2 VC cells after losartan and AngII treatment. (**n**) C6 VC cells for the co-culture between VN BV2 cells and C6 VC cells after losartan and AngII treatment. 10000 events analyzed.

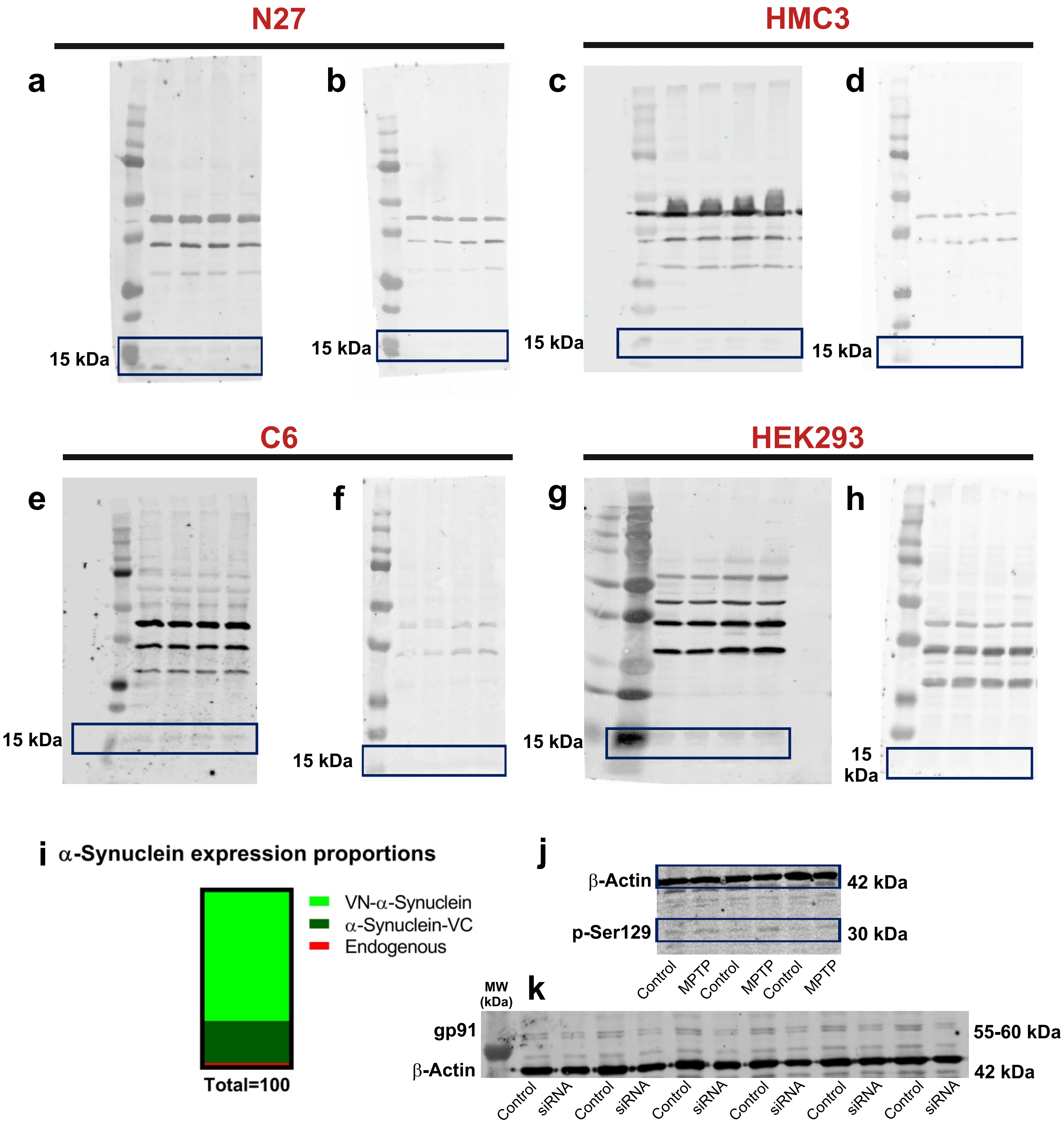

Supplementary Figure 8. Endogenous levels of α-synuclein are very low or even not detectable in western blot membrane (**a**-**h**). Full length western blot membranes showing the presence or absence of endogenous α-synuclein (blue rectangles). (**i**) Expression percentages of VN-α-synuclein (73.78 ± 7.39), α-synuclein-VC (23.88 ± 7.65) and endogenous α-synuclein (2.33 ± 1.38).In the membranes where it can be detected, endogenous α-synuclein levels are consistently below 4% of total α-synuclein. (**j**) Representative western blot membrane of the data presented in figure 6 C. The membrane shows the different expression of α-synuclein phosphorylated at the serine 129 between saline and MPTP-treated mice. (**k**) Western blot membrane showing the decrease in gp91 expression due to SiRNA treatment.

Supplementary Tables

Supplementary Table 1 Average flow cytometry values for the transfected cell lines shown in supplementary figure 4

	N27	*C6*	*HMC3*	*HEK293*
**Control**	3.97 ± 0.65	28.56 ± 1.388	40.67 ± 3.232	63.46 ± 8.013
**AngII**	3.68 ± 0.139	25.51 ± 2.486	44.09 ± 0.6891	62.27 ± 10.46
**Losartan**	4.097 ± 0.668	29.6 ± 1.181	42.98 ± 2.194	56.54 ± 9.963
**Losartan + Ang II**	3.91 ± 0.623	25.84 ± 1.703	47.77 ± 5.488	61.26 ± 9.124

percentage of cells above 100.000 random fluorescence units, mean ± SEM

Supplementary Table 2 Average flow cytometry values for the negative controls and transfected cell lines shown in supplementary figure 3

	N27	*C6*	*BV2*	*HEK293*
**Negative control**	0	0	0	0
**Vector**	0.019 ± 0.013	0.045 ± 0.039	0.006 ± 0.006	0.005 ± 0.005
**Positive control**	10.13 ± 3.85	10.396 ± 2.145	8.804 ± 3.05	57.572 ± 4.7

percentage of cells above 100.000 random fluorescence units, mean ± SEM

Supplementary Table 3 Average flow cytometry values for the experiments shown in supplementary figures 5, 6, and 7

N27 VN + N27 VC	*N27 VC + N27 VN*	*N27 VN + N27 VC + AngII*	*N27 VC + N27 VN + AngII*
0.888 ± 0.11	2.24 ± 0.467	1.04 ± 0.257	2.822 ± 0.519
N27 VN + BV2 VC	*N27 VC + BV2 VN*	*N27 VN + BV2 VC + AngII*	*N27 VC + BV2 VN + AngII*
0.53 ± 0.204	0.842 ± 0.35	0.452 ± 0.12	0.18 ± 0.076
N27 VN + C6 VC	*N27 VC + C6 VN*	*N27 VN + BV2 VC + AngII*	*N27 VN + BV2 VC + AngII*
0.287 ± 0.084	0.175 ± 0.03	0.248 ± 0.103	0.172 ± 0.065
BV2 VN + BV2 VC	*BV2 VC + BV2 VN*	*BV2 VN + BV2 VC + AngII*	*BV2 VC + BV2 VN + AngII*
0.36 ±0.07	0.307 ± 0.076	0.07 ± 0.006	1.928 ± 0.2375
BV2 VN + N27 VC	*BV2 VC + N27 VN*	*BV2 VN + N27 VC + AngII*	*BV2 VC + N27 VN + AngII*
0.557 ± 0.264	0.68 ± 0.177	1.537 ± 0.448	1.862 ± 0.5233
BV2 VN + C6 VC	*BV2 VC + C6 VN*	*BV2 VN + C6 VC + AngII*	*BV2 VC + C6 VN + AngII*
0.138 ± 0.059	0.203 ± 0.069	2.655 ± 0.48	6.682 ± 0.615
C6 VN + C6 VC	*C6 VC + C6 VN*	*C6 VN + C6 VC + AngII*	*C6 VC + C6 VN + AngII*
0.115 ± 0.05	0.1533 ± 0.049	6.938 ± 2.637	5.568 ± 1.379
C6 VN + N27 VC	*C6 VC + N27 VN*	*C6 VN + N27 VC + AngII*	*C6 VC + N27 VN + AngII*
1.78 ± 0.5	1.395 ± 0.129	0.708 ± 0.074	1.503 ± 0.354
C6 VN + BV2 VC	*C6 VC + BV2 VN*	*C6 VN + BV2 VC + AngII*	*C6 VC + BV2 VN + AngII*
0.51 ± 0.247	0.395 ± 0.099	0.155 ± 0.044	0.28 ± 0.094
BV2 VN + C6 VC + AngII + Los	*BV2 VC + C6 VN + AngII + Los*	*C6 VN + BV2 VC + AngII + Los*	*C6 VC + BV2 VN + AngII + Los*
0.753 ± 0.12	0.908 ± 0.192	0.47 ± 0.105	0.33 ± 0.041
BV2 VN + N27 VC + AngII + Los	*BV2 VC + N27 VN + AngII + Los*	*N27 VN + BV2 VC + AngII + Los*	*N27 VC + BV2 VN + AngII + Los*
0.715 ± 0.093	0.582 ± 0.12	0.512 ± 0.186	0.622 ± 0.177

percentage of cells above 98000 or 100000 random fluorescence units, mean ± SEM

Legends for movie files

Supplementary movie 1. Tridimensional projection of a control mouse striatal section immunolabeled for α-synuclein and stained for thioflavin S. The video shows very low levels of endogenous α-synuclein (red immunofluorescence) and thioflavin S positive proteins (green fluorescence).

Supplementary movie 2. Tridimensional projection of a MPTP-treated mouse striatal section immunolabeled for α-synuclein and stained for thioflavin S. This projection shows the presence of endogenous α-synuclein (red immunofluorescence) and the presence of thioflavin S positive proteins (green areas). Colocalization of red and green (orange color) reveals the presence of α-synuclein aggregates, which can be observed in striatal terminals, presumably dopaminergic terminals.

Supplementary movie 3. Tridimensional projection of a control mouse substantia nigra section immunolabeled for α-synuclein and stained for thioflavin S. The projection shows very low levels of endogenous α-synuclein (red immunofluorescence) and thioflavin S positive proteins (green fluorescence).

Supplementary movie 4. Tridimensional projection of a MPTP-treated mouse nigral section immunolabeled for α-synuclein and stained for thioflavin. This projection shows the presence of endogenous α-synuclein (red immunofluorescence) and the presence of thioflavin S positive proteins (green areas). Colocalization of red and green (orange color) shows the presence of α-synuclein aggregates in cells.

Supplementary movie 5. C6 astroglial cell expressing VN-α-synuclein capturing α-synuclein-VC released from N27 dopaminergic neurons. Presence of green fluorescence indicates the interaction between VN-α-synuclein and α-synuclein-VC inside a C6 cell. As α-synuclein-VC is expressed only by N27 cells in this experiment, the C6 cell emitting fluorescence must have captured α-synuclein-VC released by N27 cells. C6 and N27 cells were co-cultured via transwell for 72 hours. C6 cells were filmed during the whole period. Each second from the video represents three hours in real time. Only the last 48 hours are shown in the video.

Full length blots from Figure 2

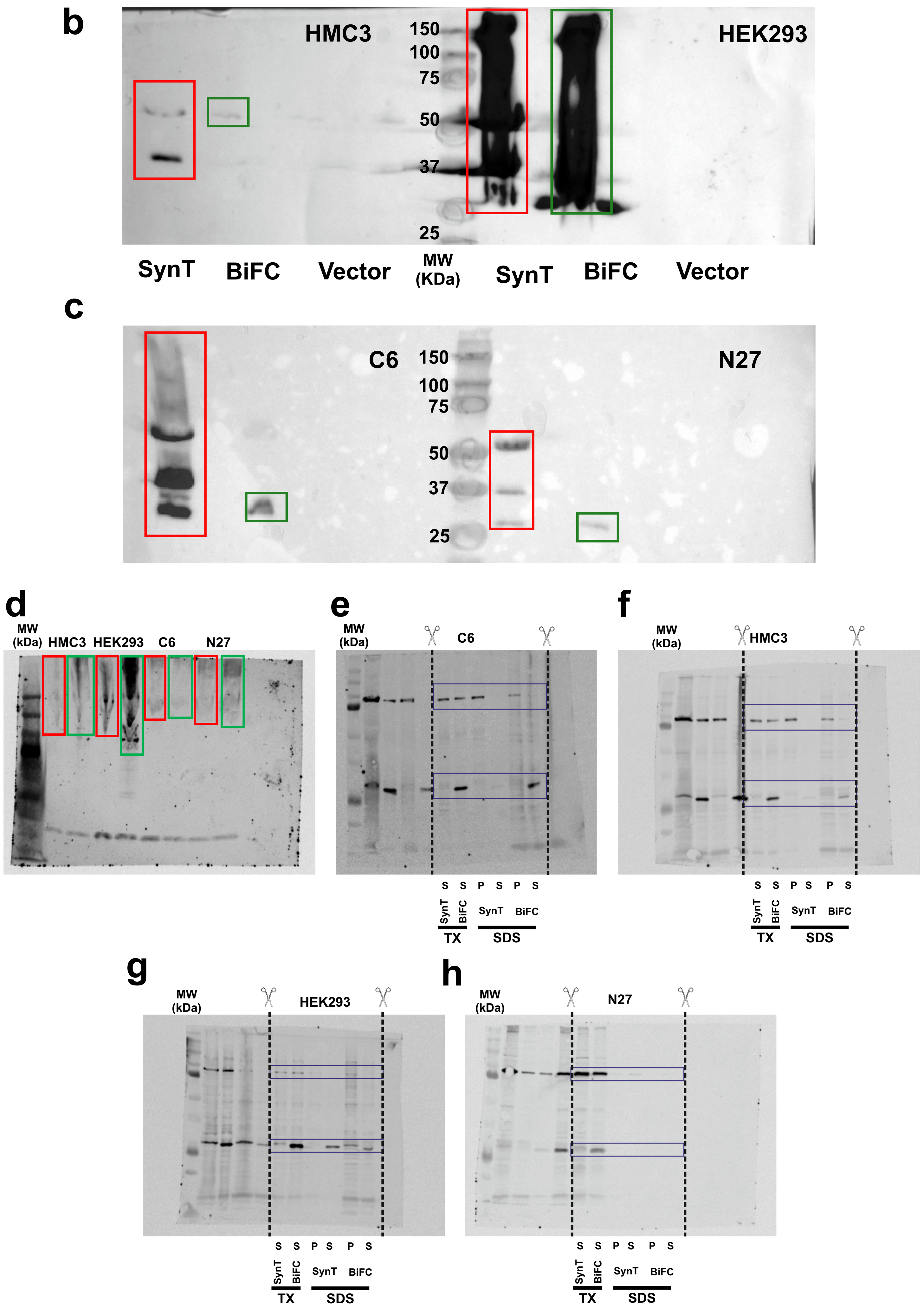

Full length blots from Figure 4

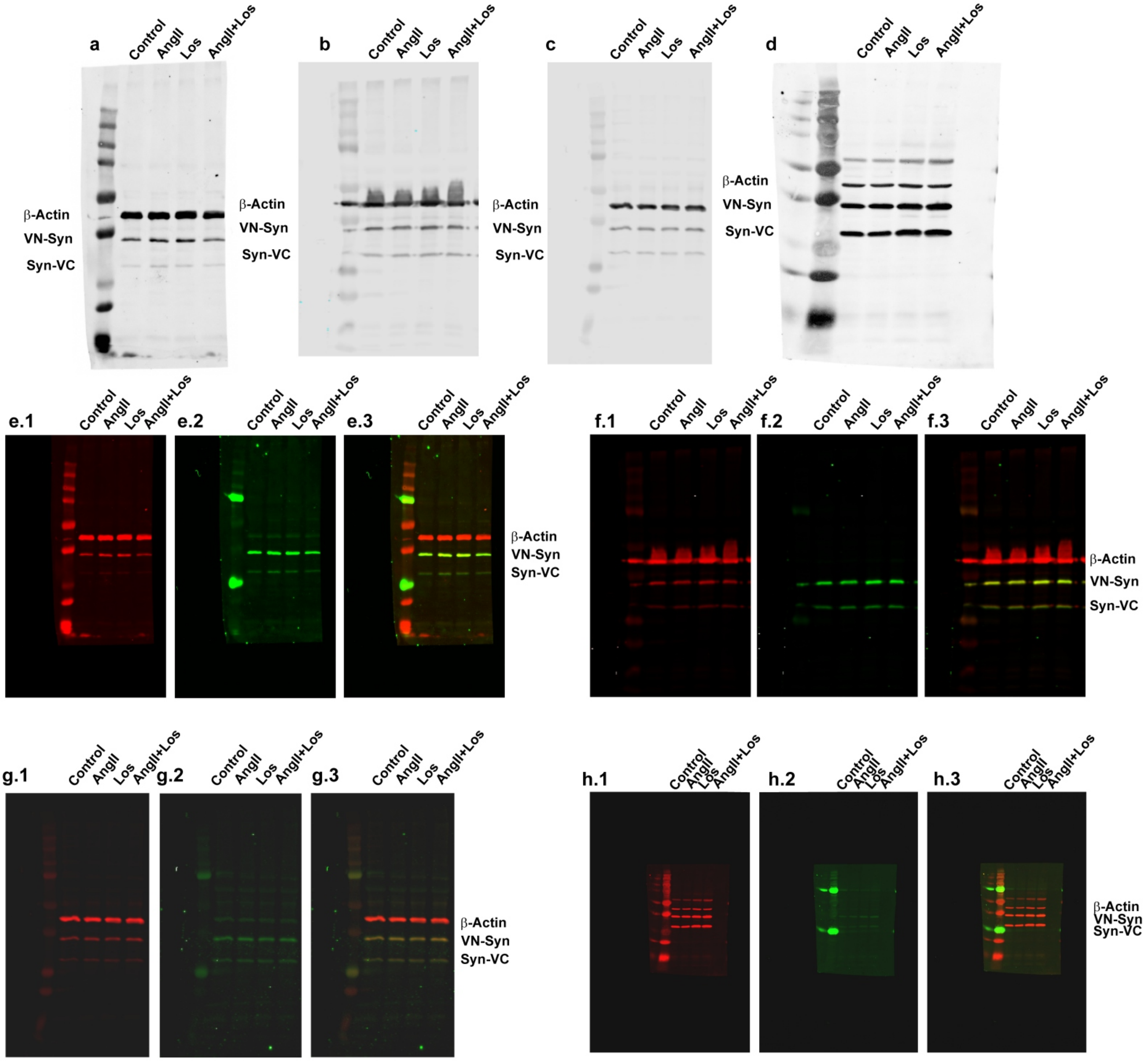

Full length blots from Figure 4. (**a-d**) Full length blots of the cropped images from figures 4 A-D. (**e-h**) Full length blots of the cropped images from figures 4 e-h, both fluorescence channels and the merged images are shown.

Full length blots from Figure 6

c

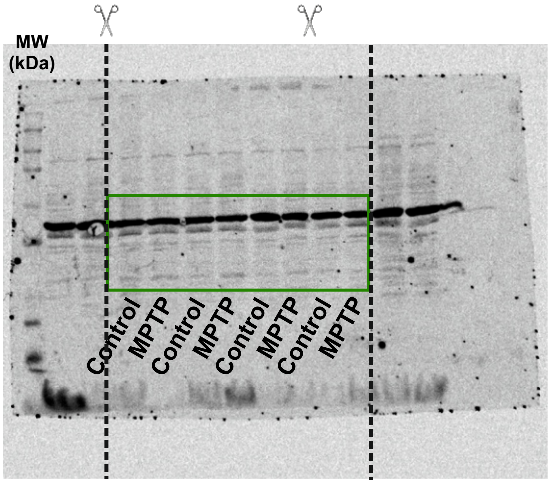

d

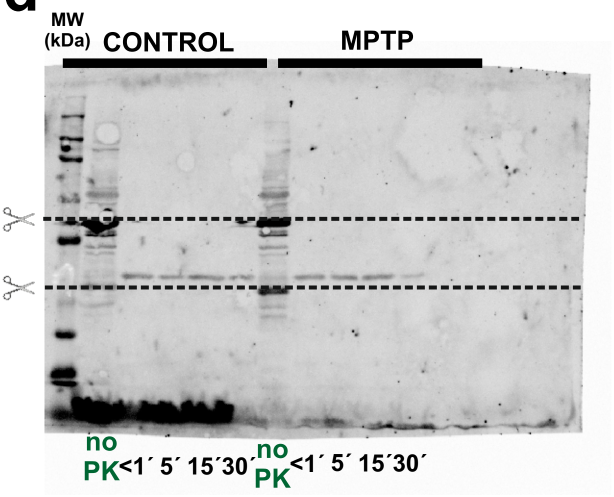

e

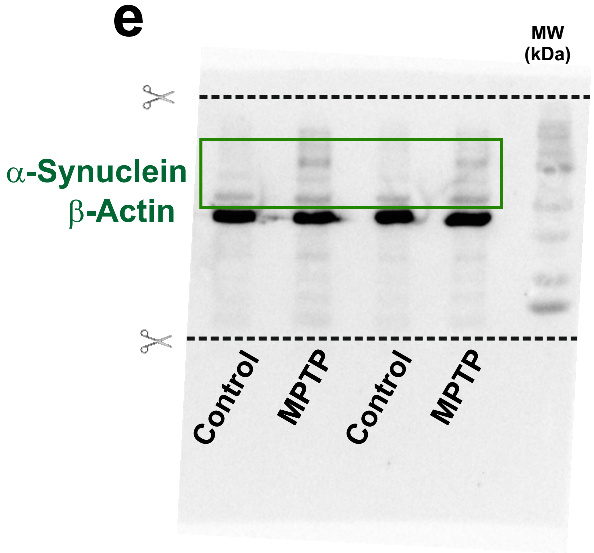

Full length blots from Figure S8

j

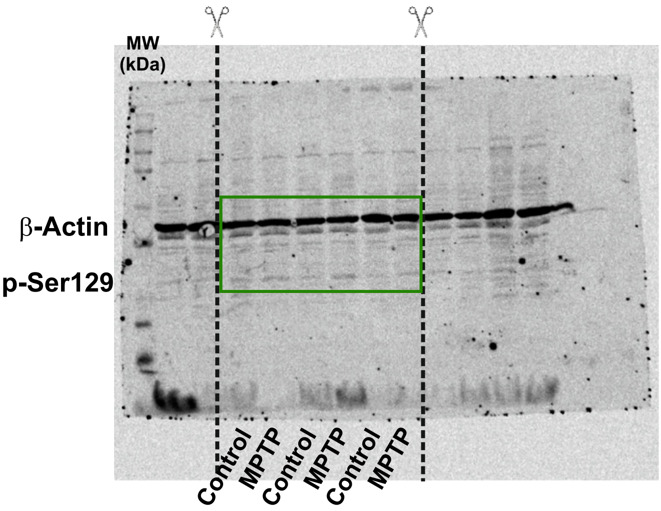

k

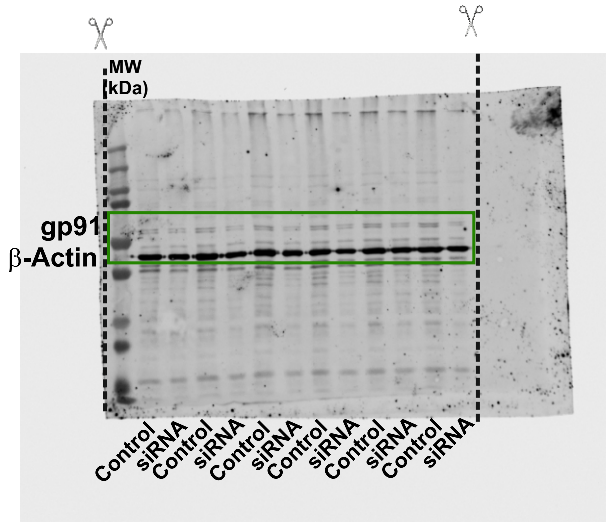

Supplement: Supplementary file 1 — Supplementary information [file 41531_2024_650_MOESM1_ESM.pdf]
