# Supplementary material for: Angiotensin type 1 receptor activation promotes neuronal and glial alpha-synuclein aggregation and transmission
Source: NPJ Parkinsons Dis. 2024 Feb 17;10:37. doi: 10.1038/s41531-024-00650-0 (PMC10874459; doi:10.1038/s41531-024-00650-0)
Supplement: Supplementary file 2 — Checklist [file 41531_2024_650_MOESM2_ESM.pdf]

nature portfolio

Corresponding author(s): Jose Luis Labandeira-Garcia
Antonio Dominguez-Meiide

Last updated by author(s): Oct 15, 2023

Reporting Summary

Nature Portfolio wishes to improve the reproducibility of the work that we publish. This form provides structure for consistency and transparency in reporting. For further information on Nature Portfolio policies, see our Editorial Policies and the Editorial Policy Checklist.

Statistics

For all statistical analyses, confirm that the following items are present in the figure legend, table legend, main text, or Methods section.

n/a	Confirmed	
☐	☒	The exact sample size (*n*) for each experimental group/condition, given as a discrete number and unit of measurement
☐	☒	A statement on whether measurements were taken from distinct samples or whether the same sample was measured repeatedly
☐	☒	The statistical test(s) used AND whether they are one- or two-sided<br>*Only common tests should be described solely by name; describe more complex techniques in the Methods section.*
☐	☒	A description of all covariates tested
☐	☒	A description of any assumptions or corrections, such as tests of normality and adjustment for multiple comparisons
☐	☒	A full description of the statistical parameters including central tendency (e.g. means) or other basic estimates (e.g. regression coefficient) AND variation (e.g. standard deviation) or associated estimates of uncertainty (e.g. confidence intervals)
☒	☐	For null hypothesis testing, the test statistic (e.g. *F*, *t*, *r*) with confidence intervals, effect sizes, degrees of freedom and *P* value noted<br>*Give P values as exact values whenever suitable.*
☒	☐	For Bayesian analysis, information on the choice of priors and Markov chain Monte Carlo settings
☒	☐	For hierarchical and complex designs, identification of the appropriate level for tests and full reporting of outcomes
☒	☐	Estimates of effect sizes (e.g. Cohen's *d*, Pearson's *r*), indicating how they were calculated

Our web collection on statistics for biologists contains articles on many of the points above.

Software and code

Policy information about availability of computer code

Data collection	- BD CSampler C6 software (Accuri): software for flow cytometry data adquisition and analysis<br>- Leica application suite X LASX (Leica): software for microscopy picture adquisition and analysis<br>- ImageJ 1.51j8 (NIH, USA): picture analysis<br>- Molecular Imager ChemiDoc MP imaging System (BioRad): fluorescence detection system for Western Blot analysis<br>- Molecular Imager ChemiDoc XRS System (BioRad): chemiluminescence detection system for Western Blot analysis
Data analysis	-GraphPad Prism 8 software (GraphPad Inc., San Diego, CA, USA): statistical analyses of in vitro and animal experiments and to create scatter dot plot graphs

For manuscripts utilizing custom algorithms or software that are central to the research but not yet described in published literature, software must be made available to editors and reviewers. We strongly encourage code deposition in a community repository (e.g. GitHub). See the Nature Portfolio guidelines for submitting code & software for further information.

Data

Policy information about availability of data

All manuscripts must include a data availability statement. This statement should provide the following information, where applicable:

- Accession codes, unique identifiers, or web links for publicly available datasets
- A description of any restrictions on data availability
- For clinical datasets or third party data, please ensure that the statement adheres to our policy

The authors declare that the data supporting the findings of this study are available within the article

Research involving human participants, their data, or biological material

Policy information about studies with human participants or human data. See also policy information about sex, gender (identity/presentation), and sexual orientation and race, ethnicity and racism.

Reporting on sex and gender	*Use the terms sex (biological attribute) and gender (shaped by social and cultural circumstances) carefully in order to avoid confusing both terms. Indicate if findings apply to only one sex or gender; describe whether sex and gender were considered in study design; whether sex and/or gender was determined based on self-reporting or assigned and methods used. Provide in the source data disaggregated sex and gender data, where this information has been collected, and if consent has been obtained for sharing of individual-level data; provide overall numbers in this Reporting Summary. Please state if this information has not been collected. Report sex- and gender-based analyses where performed, justify reasons for lack of sex- and gender-based analysis.*
Reporting on race, ethnicity, or other socially relevant groupings	*Please specify the socially constructed or socially relevant categorization variable(s) used in your manuscript and explain why they were used. Please note that such variables should not be used as proxies for other socially constructed/relevant variables (for example, race or ethnicity should not be used as a proxy for socioeconomic status). Provide clear definitions of the relevant terms used, how they were provided (by the participants/respondents, the researchers, or third parties), and the method(s) used to classify people into the different categories (e.g. self-report, census or administrative data, social media data, etc.) Please provide details about how you controlled for confounding variables in your analyses.*
Population characteristics	*Describe the covariate-relevant population characteristics of the human research participants (e.g. age, genotypic information, past and current diagnosis and treatment categories). If you filled out the behavioural & social sciences study design questions and have nothing to add here, write "See above."*
Recruitment	*Describe how participants were recruited. Outline any potential self-selection bias or other biases that may be present and how these are likely to impact results.*
Ethics oversight	*Identify the organization(s) that approved the study protocol.*

Note that full information on the approval of the study protocol must also be provided in the manuscript.

Field-specific reporting

Please select the one below that is the best fit for your research. If you are not sure, read the appropriate sections before making your selection.

☒ Life sciences ☐ Behavioural & social sciences ☐ Ecological, evolutionary & environmental sciences

For a reference copy of the document with all sections, see nature.com/documents/nr-reporting-summary-flat.pdf

Life sciences study design

All studies must disclose on these points even when the disclosure is negative.

Sample size	The mininmal numbers of animals per group was estimated has been estimated taking into account the principle of replacement, refinement and reduction (RRR) following previous works from our lab
Data exclusions	No data were excluded for the analysis
Replication	Experiments were replicated at least 6 times
Randomization	Animals and cellular cultures were randomly selected for each group
Blinding	In animal and in vitro experiments researchers that obtained the samples were also different to those that processed them and were blinded to their origin

Reporting for specific materials, systems and methods

We require information from authors about some types of materials, experimental systems and methods used in many studies. Here, indicate whether each material, system or method listed is relevant to your study. If you are not sure if a list item applies to your research, read the appropriate section before selecting a response.

Materials & experimental systems

n/a	Involved in the study
☐	☒ Antibodies
☐	☒ Eukaryotic cell lines
☒	☐ Palaeontology and archaeology
☐	☒ Animals and other organisms
☒	☐ Clinical data
☒	☐ Dual use research of concern
☒	☐ Plants

Methods

n/a	Involved in the study
☒	☐ ChIP-seq
☐	☒ Flow cytometry
☒	☐ MRI-based neuroimaging

Antibodies

Antibodies used

Rabbit monoclonal anti α-synuclein antibody MJFR1 (ab138501, Abcam)
Goat polyclonal anti V5 antibody (ab9137, Abcam)
Mouse monoclonal α-synuclein LB509 (ab27766, Abcam)
Mouse monoclonal antibodies against α-synuclein (Syn 211) (ab80627, Abcam)
Rabbit polyclonal phosphorylated α-synuclein S129 (ab59264, Abcam)
Mouse monoclonal Glial Fibrillary Acidic Protein (GFAP) (MAB360 EMD, Millipore)
Rabbit monoclonal ionized calcium-binding adapter molecule 1 EPR6136 (IBA1) (ab178680, Abcam)
Mouse monoclonal antibodies against Tyrosine Hydroxylase (TH) (T2928, Sigma Aldrich)
Mouse monoclonal antibody β-actin (A2228, Sigma)
Polyclonal Alexa Fluor 488-conjugated donkey anti-rabbit IgG (A-21206, Thermo Fisher Scientific)
Polyclonal Alexa Fluor 568-conjugated donkey anti-rabbit IgG (A10042, Thermo Fisher Scientific)
Polyclonal Alexa Fluor 633-conjugated donkey anti-goat IgG (A-21082, Thermo Fisher Scientific).
Polyclonal Alexa fluor 568 conjugated-donkey anti rabbit (A10042, Thermo Fisher Scientific)
Polyclonal Alexa fluor 647 conjugated-donkey anti mouse (A-31571, Thermo Fisher Scientific)
Polyclonal Alexa fluor 568 conjugated-donkey anti mouse (A10037, Thermo Fisher Scientific)
Polyclonal Alexa fluor 647 conjugated-donkey anti rabbit (A-31573, Thermo Fisher Scientific)
Donkey anti mouse IRDye 680LT (926-68022, LI-COR).
Donkey anti rabbit IRDye 800CW (926-32213, LI-COR).

Validation

Rabbit monoclonal anti α-synuclein antibody MJFR1 (Abcam Cat# ab138501, RRID:AB_2537217; 1:1500)
Goat polyclonal anti V5 antibody (Abcam Cat# ab9137, RRID:AB_307037; 1:3000)
Mouse monoclonal α-synuclein LB509 (Abcam Cat# ab27766, RRID:AB_727020; 1:500)
Mouse monoclonal anti α-synuclein (Syn 211) (Abcam Cat# ab80627, RRID:AB_1603277; 1:2000)
Rabbit polyclonal phosphorylated α-synuclein S129 (Abcam Cat# ab59264, RRID:AB_2270761; 1:2000)
Mouse monoclonal Glial Fibrillary Acidic Protein (GFAP) (Millipore Cat# MAB360, RRID:AB_11212597; 1:1000)
Rabbit monoclonal ionized calcium-binding adapter molecule 1 EPR6136 (IBA1) Abcam Cat# ab178680, RRID:AB_2755129; 1:100)
Mouse monoclonal antibodies against Tyrosine Hydroxylase (TH) (Sigma-Aldrich Cat# T2928, RRID:AB_477569;1:1000)
Mouse monoclonal antibody β-actin (Sigma-Aldrich Cat# A2228, RRID:AB_476697; 1:10000)
Polyclonal Alexa Fluor 488-conjugated donkey anti rabbit IgG (Thermo Fisher Scientific Cat# A-21206, RRID:AB_2535792; 1:200)
Polyclonal Alexa Fluor 568-conjugated donkey anti rabbit IgG (Thermo Fisher Scientific Cat# A10042, RRID:AB_2534017; 1:200)
Polyclonal Alexa Fluor 633-conjugated donkey anti goat IgG (Thermo Fisher Scientific Cat# A-21082, RRID:AB_2535739; 1:200).
Polyclonal Alexa fluor 568 conjugated-donkey anti rabbit (Thermo Fisher Scientific Cat# A10042, RRID:AB_2534017; 1:100)
Polyclonal Alexa fluor 647 conjugated-donkey anti mouse (Thermo Fisher Scientific Cat# A-31571, RRID:AB_162542; 1:100)
Polyclonal Alexa fluor 568 conjugated-donkey anti mouse (Thermo Fisher Scientific Cat# A10037, RRID:AB_2534013; 1:100)
Polyclonal Alexa fluor 647 conjugated-donkey anti rabbit (Thermo Fisher Scientific Cat# A-31573, RRID:AB_2536183; 1:100)
Polyclonal donkey anti mouse IRDye 680LT (LI-COR Biosciences Cat# 926-68022, RRID:AB_10715072; 1:20000)
Polyclonal donkey anti rabbit IRDye 800CW (LI-COR Biosciences Cat# 926-32213, RRID:AB_621848; 1:20000).

Eukaryotic cell lines

Policy information about cell lines and Sex and Gender in Research

Cell line source(s)

Dopaminergic N27 cell line derived from rat female mesencephalic
tissue (SCC048; Millipore), BV2 cell line from murine microglia (ABC-TC212S; AcceGen), HMC3 cell line from human microglia (CRL-3304; ATCC) and C6 glial cell line from rat brain (CCL-107; ATCC) and HEK293 cell line from human embryonic kidney (CRL-1573; ATCC)

Authentication	Not applicable because they are comercial lines
Mycoplasma contamination	Confirmed that all cell lines tested negative for mycoplasma contamination
Commonly misidentified lines (See ICLAC register)	Not applicable

Animals and other research organisms

Policy information about studies involving animals; ARRIVE guidelines recommended for reporting animal research, and Sex and Gender in Research

Laboratory animals	-For in vivo experiments, we used young male C57BL6/J mice (8 week-old) obtained from the Center of Experimental Biomedicine of Galicia (CEBEGA)
Wild animals	The study did not involve wild animals
Reporting on sex	All animals used are male to avoid the effects that estrogens reportedly have in neuroprotection and alpha-synuclein aggregation, which may hide the effects investigated in the present study.
Field-collected samples	The study did not involve samples collected from the field
Ethics oversight	-Ethics Committee at the University of Santiago de Compostela (protocol 14715012/2021/012; last revision 16/04/2021)<br>-European Communities Council Directive 2010/63/EU 145 and Directive

Note that full information on the approval of the study protocol must also be provided in the manuscript.

Flow Cytometry

Plots

Confirm that:

- [x] The axis labels state the marker and fluorochrome used (e.g. CD4-FITC).
- [x] The axis scales are clearly visible. Include numbers along axes only for bottom left plot of group (a 'group' is an analysis of identical markers).
- [ ] All plots are contour plots with outliers or pseudocolor plots.
- [x] A numerical value for number of cells or percentage (with statistics) is provided.

Methodology

Sample preparation	All cells used in flow cytometry are commercial cell lines (neuronal N27, microglial BV2 and HMC3, astroglial C6 and kidney HEK293). Cells were first trypsinized to lift them and then they were neutralized with FBSi medium. The cell suspension was centrifuged, the supernatant discarded, and the pellet was resuspended in phosphate buffered saline (PBS) + 5 % FBSi
Instrument	BD Accuri C6 Plus Personal Flow Cytometer
Software	The software used for flow cytometry data adquisition and analysis was BD CSampler C6 software (Accuri)
Cell population abundance	After the preliminary FSC/SCC gating the analyzed populations were the following percentages of the total number of events:<br>53.36% ± 7.37% SEM for N27 cells<br>66.16% ± 8.96% SEM for BV2 cells<br>36.37% ± 2.73% SEM for HMC3 cells<br>64.28% ± 2.53% SEM for C6 cells<br>46.86% ± 4.04% SEM for HEK293
Gating strategy	- Cell population was chosen from a preliminary FSC/SCC gating.<br>- Positive cells were defined as cells with a venus fluorescence intensity above 100.000 to 120.000 RFU, based on the values of fluorescence intensity of the negative and positive controls.

- [x] Tick this box to confirm that a figure exemplifying the gating strategy is provided in the Supplementary Information.
